# Workplace Well-Being in Industry 5.0: A Worker-Centered Systematic Review

**DOI:** 10.3390/s24175473

**Published:** 2024-08-23

**Authors:** Francesca Giada Antonaci, Elena Carlotta Olivetti, Federica Marcolin, Ivonne Angelica Castiblanco Jimenez, Benoît Eynard, Enrico Vezzetti, Sandro Moos

**Affiliations:** 1 Department of Management and Production Engineering, Politecnico di Torino, Corso Duca degli Abruzzi 24, 10129 Turin, Italy; 2 Department of Mechanical Systems Engineering, Université de Technologie de Compiègne, Centre Pierre Guillaumat, BP 60319, Rue du Docteur Schweitzer, Cedex, F-60203 Compiègne, France

**Keywords:** workplace well-being, physical ergonomics, cognitive ergonomics, industry 5.0, human–robot interaction, human–machine interaction

## Abstract

The paradigm of Industry 5.0 pushes the transition from the traditional to a novel, smart, digital, and connected industry, where well-being is key to enhance productivity, optimize man–machine interaction and guarantee workers’ safety. This work aims to conduct a systematic review of current methodologies for monitoring and analyzing physical and cognitive ergonomics. Three research questions are addressed: (1) which technologies are used to assess the physical and cognitive well-being of workers in the workplace, (2) how the acquired data are processed, and (3) what purpose this well-being is evaluated for. This way, individual factors within the holistic assessment of worker well-being are highlighted, and information is provided synthetically. The analysis was conducted following the PRISMA 2020 statement guidelines. From the sixty-five articles collected, the most adopted (1) technological solutions, (2) parameters, and (3) data analysis and processing were identified. Wearable inertial measurement units and RGB-D cameras are the most prevalent devices used for physical monitoring; in the cognitive ergonomics, and cardiac activity is the most adopted physiological parameter. Furthermore, insights on practical issues and future developments are provided. Future research should focus on developing multi-modal systems that combine these aspects with particular emphasis on their practical application in real industrial settings.

## 1. Introduction

The digital transition opened up by the concept of Industry 5.0 [1] is leading to the necessity of a further step in the development of new manufacturing systems. In fact, if in the last decade the production has been boosted to smart, connected, self-controlling manufacturing (Industry 4.0), the future of the industry is inseparable from the development of a sustainable, resilient, and human-centered production system, given the utmost importance of the human factor [2] (Industry 5.0). The adoption of this approach in the ongoing industrial revolution raises questions about the coexistence between the technology-centricity of the former and the human-centricity of the latter [3]. Industry 5.0 represents a departure from its predecessor in that it places an emphasis on the harmonious integration of advanced technologies with human-centric values. This approach is designed to create environments where human well-being, rather than mere technological advancement, is the focal point of innovation. This paradigm shift addresses the growing need to balance automation with human involvement, ensuring that technological progress enhances rather than diminishes human roles in manufacturing [4]. From this viewpoint, the “age of augmentation” appears as that of the reconciliation between technology-driven and value-driven paradigms of the factory of the future [5], in which digital technologies, such as extended reality (XR), artificial intelligence (AI), and collaborative robotics, will develop in a human–machine co-evolution, built upon inclusiveness and sustainability, to reach high performances throughout an adaptive relationship between technology and human. The specific issue under investigation in this research is the optimal integration of these advanced technologies within the context of Industry 5.0, ensuring the continued primacy of worker well-being in industrial processes [3]. In this sense, Industry 5.0 is intended to pursue a human-centric adaptive manufacturing system [6,7,8,9] through a wide employment of acquisition technologies aimed to monitor and acquire data related to workers that are subsequently analyzed through the usage of AI techniques. In this scenario, machine learning (ML) [10] and deep learning (DL) [11] algorithms proved to be suitable approaches to obtain cutting-edge results in terms of classification performances, supporting workers both from efficiency and safety perspectives [12,13,14] and allowing the designers to shift from a less demanding paradigm (ML) to a more challenging, versatile, and potentially performing one (DL).

In light of this new wave in the production panorama, a reasonable question arises: how can the workplace be re-engineered to reach the human-centric paradigm of the factory of the future? Indeed, knowledge about the actual perceived well-being of workers on the workplace is crucial to understand how to design the workplace of the future. In this sense, “holistic ergonomics” [15] is an approach to the design and arrangement of workspaces and systems that takes into account the entire well-being of individuals within a given environment, gathering the key factors into the following categories: physical, cognitive, emotional, social, and organizational [16,17]. Attention toward ergonomics often focuses on physical factors such as posture, lighting and equipment design to optimize efficiency and prevent physical strain or injury, but it neglects the other aspects of holistic ergonomics, namely cognitive ergonomics and social ergonomics, which can affect a person’s overall well-being and performance. This study, therefore, seeks to address the current research gap by examining the dual aspects of physical and cognitive ergonomics. These are the two elements of holistic ergonomics that can directly influence the design (or re-design) of the human-centered workplace advocated by Industry 5.0. Physical ergonomics involves those aspects of ergonomics [18] addressing factors such as seating, workstation layout, and tools to promote comfortable and efficient physical work. Cognitive ergonomics [19] focuses on mental processes, including perception, memory, and decision making. Designing work environments that support cognitive functions can improve overall productivity and reduce mental fatigue.

In light of the aforementioned considerations, the aim of this work is to conduct a systematic review of the state-of-the-art technologies and methodologies used to analyze workers’ well-being from these two viewpoints. This review employs rigorous methodology to examine existing literature, using a structured approach to identify, evaluate, and synthesize relevant studies, ensuring that the findings are both comprehensive and applicable to real-world industrial settings [20]. In fact, it is core to monitor and analyze the worker’s well-being in the workplace as an individual before considering the interactions in terms of social dynamics involving collaboration, communication, teamwork, and also company policies and procedures, particularly in the industrial domain. Since analyzing workers’ well-being is an increasingly cross-disciplinary task, the review includes studies about (1) the acquisition, tracking, and recognition of workers’ posture and movements related to the physical point of view, and (2) studies on the physiological measurements to assess the mental workload and work-related mental stress. This way, the wide panorama of existing solutions can be captured, and researchers on this topic can receive essential information on the strengths and weaknesses of current solutions on which to base future studies for the materialization of the human-centered factory promoted by Industry 5.0.

The objective of this literature review can be resumed in three research questions (RQs):

- RQ1: What are the state-of-the-art technologies currently adopted for the assessment of both physical and cognitive well-being in industrial workplaces?
- RQ2: What advanced data processing methods, including those belonging to the field of AI, are employed to interpret the data acquired from these well-being assessments?
- RQ3: What are the principal objectives of assessing the well-being of workers in the context of Industry 5.0, and how do these assessments contribute to enhancing productivity, safety, and human–machine interaction in smart factories?

By addressing these research questions, this study not only elucidates the current state of the art in the assessment of worker well-being but also paves the way for future developments in this field, offering insights of value to both academic researchers and industry practitioners.

The study is structured as follows: the employed methodology is described in the next Section 2; then, the review results are presented Section 3 and debated in Section 4; finally, conclusions are drawn in Section 5.

## 2. Methodological Analysis

**Figure 1:**
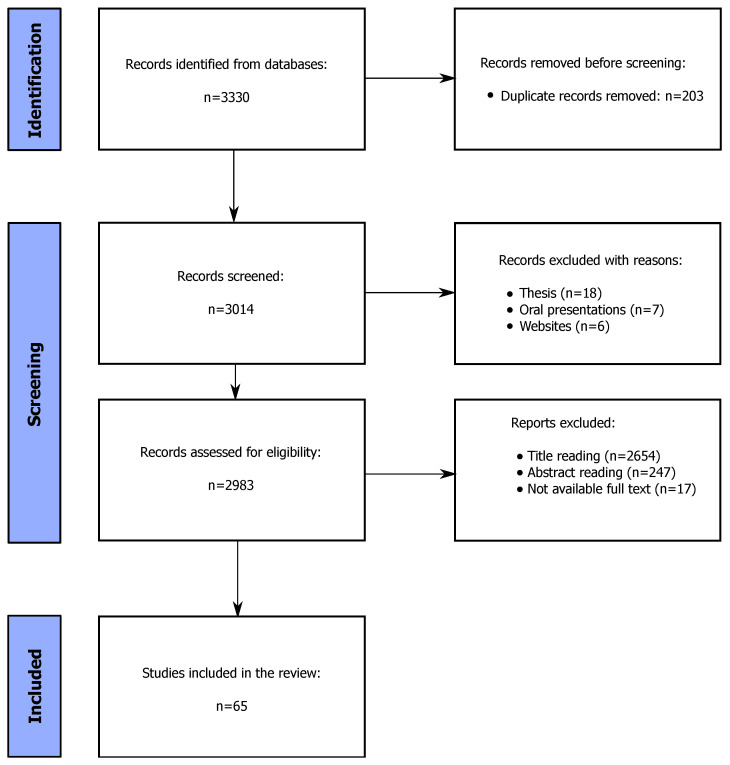
PRISMA flowchart.

The analysis was conducted following the PRISMA 2020 statement guidelines [20] (Figure 1).

A set of criteria was established to evaluate the eligibility of the papers:

1. Databases used for the search: Google Scholar and Scopus;
2. Only articles from indexed, peer-reviewed journals were selected;
3. Publication year: papers published before 2016 were excluded;
4. Context of the application: only papers involving industrial/productive contexts were included;
5. An appropriate combination of keywords, detailed below, has been used for the paper selection.

Due to the lack of studies integrating the physical and cognitive perspectives, the investigation follows a two-fold approach, considering both the articles focused on the physical perspective and the papers about evaluation related to the cognitive point of view. This way, a comprehensive analysis of the assessment of workers’ well-being in the workplace has been drawn. The following keywords were considered for dealing with the physical perspective: artificial intelligence, machine learning, deep learning combined with body tracking, body recognition, motion capture, gesture recognition, postural monitoring, posture monitoring, and ergonomics, each of them combined with manufacturing, assembly line, industrial (for greater transparency, see Table 1). Similarly, the following keywords were adopted to deepen the cognitive perspective: mental ergonomics, mental workload, work-related stress, and cognitive ergonomics, which, in turn, were combined with assembly line, manufacturing, and workplace design (for greater transparency, see Table 2).

The resulting sixty-five articles have been assessed by four investigators: a mechanical investigator, two biomedical investigators, and a computer science engineer. The diversity of the investigators’ backgrounds has been core to taking into consideration the different expertise necessary for the design and implementation of these solutions. In fact, these solutions require the employment of task-specific technology (acquisition systems, wearable sensors, etc.) to gather different types of data depending on the target analysis. These data must be classified, typically through AI techniques, and interpreted to correctly provide feedback to the users that could support the working activity (e.g., posture correction). This process of selection of the articles allowed to assess the presence of potential bias in the articles, excluding those that did not meet with the approval of all four investigators.

At the end of the paper selection process, forty-two articles related to the physical perspective and twenty-three articles related to the cognitive perspective have been selected. The research revealed a very low level of overlap between the two sets of keywords, which was evidenced by the fact that very few papers covered both physical and cognitive aspects, resulting in an inevitable need to integrate these two perspectives in order to draw a comprehensive review on the workers’ well-being assessment on the workplace.

The categories according to which the selected articles have been classified are the following ones:

- Authors;
- Year;
- Monitored activity: physical monitoring task or adopted physiological measurement;
- Data acquisition device: class of the devices employed in the examined study;
- Data acquisition device model;
- Data processing approach: machine learning (ML) or deep learning (DL). This information is particularly useful to group the studies in terms of cost–benefit ratio, recalling that DL typically provides state-of-the-art results to the cost of being equally demanding in terms of the amount of data and required computational power;
- Data processing algorithm: ML and DL are general approaches that identify a wide range of different specific algorithms;
- Ergonomics: a label to identify, eventually, if the study falls in the physical ergonomics or in the cognitive ergonomics domain;
- Standard ergonomic index or physiological measure: punctual information highlighting the adoption of a specific index in case of a study falling into the physical domain or one or multiple physiological measures in case of a study falling into the cognitive domain.

## 3. Results

In this section, the papers resulting from the review are presented. Table 3 summarizes the contents and the significant information related to the questions this review aims to investigate.

The resulting papers have been gathered according to the ergonomics branch to which they refer (Section 3.1 and Section 3.2). Moreover, in Section 3.1, papers are further grouped into three subsections in order to highlight the purpose of analyzing physical ergonomics in the considered activities. In fact, Section 3.1.1 focuses on fostering the manual processes through the analysis of the workers’ behavior in terms of gestures and posture. Section 3.1.2 highlights the importance of adopting a user-centered approach to foster man–machine interaction (MMI) both in terms of productivity and safety. Section 3.1.3 specifically explores those studies related to the physical ergonomics assessment tools, which are indexes employed to assess and improve safety, fatigue, and discomfort to preserve workers’ health and well-being. Section 3.2 collects the papers in which the workers’ well-being is considered from the viewpoint of mental and cognitive ergonomics. Papers are presented first referring to the employed acquisition technology, then to the use of artificial intelligence techniques (machine learning and deep learning) for the workers’ physical and cognitive parameters identification and classification, and lastly to the ergonomic assessment. This structure may vary depending on the information provided by the selected papers.

### 3.1. Physical Ergonomics

As explained above, in this section, the resulting papers have been grouped according to the purpose of the physical ergonomics analysis: productivity enhancement, man–machine interaction optimization, and safety support.

#### 3.1.1. Productivity Enhancement

The ratio behind the following articles relies on the idea that ergonomically designed environments and tools can contribute to increase productivity. When people are comfortable and not experiencing physical discomfort or fatigue, they are more likely to maintain focus and work efficiently.

In this sense, Grzeszick et al. [22] presented a new method for optimizing manual processes in factories and warehouses using human activity recognition (HAR). They collected data from two warehouses, focusing on order picking, using inertial measurement units (IMUs) on the wrists and torso. The data were fed into a deep neural network using temporal convolutions, allowing different sensors to be handled separately and information fused at each step. The IMU-centered CNN architecture showed improved results compared to conventional methods.

In the paper written by Moya Rueda et al. [24], the authors proposed a CNN for HAR using multi-channel time-series data from inertial sensors worn by warehouse workers. This architecture reduces asynchrony between sensors and outperforms traditional CNNs and previous approaches, with a detection accuracy of around 71%, which was justified by its theoretical advantage.

In the work by Chen et al. [26], the RGB camera and Kinect camera were used to monitor repetitive assembly operations using dynamic learning methods. The Kinect camera, equipped with RGB technology, can compute skeleton joints and track larger scenes, reducing segment misalignment issues during complex tasks [85]. These cameras use RGB data for object recognition and human pose estimation, detecting worker activity and assessing repetitive assembly movements. The YOLOv3 object detection algorithm has a 92.8% accuracy in action detection, while the convolutional pose machine (CPM) pose estimation algorithm has an 82.1% accuracy in determining repetitive assembly action timing.

The research held by Jiao et al. [27] proposed a framework for action recognition using RGB cameras in industrial workflows. It used deep-learning networks like CNNs, spatial transformer networks (STNs), and graph convolutional networks (GCNs) to extract spatial and temporal information from videos, estimate human poses, and obtain operator skeletons using YOLOv3. STN corrects skeleton images, while GCN extracts spatial and temporal information simultaneously.

Niemann et al. [33] highlighted the importance of automating the assessment of human activity in production and logistics to enhance efficiency and ergonomics. They present a dataset of picking and packing tasks with context information and introduce an activity recognition model that combines motion data and context information. This model, using optical IR marker-based motion capture (oMoCap), improves performance by feeding data into a temporal Convolutional Neural Network (tCNN) for shallow classifier prediction. Several classifiers, including decision trees (DTs), are tested, ensuring that processing human motion data requires sophisticated feature extraction models that are domain-independent.

With reference to De Feudis et al. [37], we evaluated some vision-based hand tool tracking methods for quality assessment and training in human-centric Industry 4.0. It compares four tracking systems, including Azure Kinect Body Tracking technology, which uses Microsoft’s Azure Kinect Body Tracking SDK for 3D body configuration estimation. The system tracks upper limb segments using sensors like RGB cameras, depth cameras, microphone arrays, and orientation sensors. The study explores two methods of tool tracking: direct detection and tracking of the tool itself and indirect tracking of the operator’s body joints and hands to infer tool pose. OpenPose is the most comprehensive option, offering acceptable point-to-point distance and low variability.

From these first proposed studies, and a considerable number of studies described below, it has been confirmed that DL, and in particular convolutional-based architectures, gain a not negligible role in these domains.

#### 3.1.2. Man–Machine Interaction Optimisation

In settings where humans interact with machines, physical ergonomics is essential for ensuring that interfaces, controls, and displays are designed in a way that is intuitive and minimizes the potential for errors. The following articles present solutions according to this perspective.

Pławiak et al. [21] developed a system in which the DG5 VHand glove device is designed to efficiently recognize hand gestures in body language. It consists of ten sensors, including finger flexion sensors, accelerometers, and gyroscopes. The glove interface connects to an external device via a four-wire connector. Researchers used the data to train a machine-learning approach, using the support vector machine (SVM), which achieved a sensitivity of 98.32%.

Luo et al. [23] involved the implementation of the VICON system, a combination of nine markers and three rigid plates, which is used to predict human reaching motions quickly, enabling robots to avoid interference while performing complementary tasks. The system uses an unsupervised learning algorithm to model trajectories, learning Gaussian mixture models (GMMs) to adapt robot behavior in real time. This approach is effective in human–robot collaboration (HRC). The two-layer framework updates models with new observations, distinguishing it from previous methods that rely on supervised learning and manual labeling. The system uses Gaussian mixture regression (GMR) to predict the missing path in the remaining trajectory by identifying the GMM.

In the research led by Urgo et al. [25], the authors presented a human modeling and monitoring approach to support manufacturing operations in high variability collaborative environments. It uses RGB data and hidden Markov models (HMMs) to identify errors and unsafe situations. The OpenPose library is used for operator pose estimation, enabling the real-time recognition and tracking of human body parts. The HMM mitigates uncertainties in acquired variables, making it suitable for tasks requiring pose estimation.

The research by Manitsaris et al. [28] explored the use of human-centered artificial intelligence in professional workplaces to improve collaboration, skills and work quality. RGB sensors capture and segment gesture images, enabling pose estimation and operator skeleton extraction. In real-world scenarios, an RGB-D camera extracts 3D hand positions. The HMM is implemented on RGB and RGB-D data and translated into concurrent equations using state space (SS) modeling and maximum likelihood estimation. Simulations create confidence-based spatial variance tolerance bounding boxes that are effective for gesture recognition and human motion trajectory prediction.

Xiong et al. [30] proposed a transferable two-stream CNN architecture for human action recognition in manufacturing environments. The architecture uses optical flow to extract temporal and spatial information from video images and transfer learning to transfer a model learned in one domain to another target domain.

Luipers and Richert [31] focused on improving human–robot interaction efficiency and safety by increasing the predictability and intuitiveness of cobot movements. The authors used a Microsoft Kinect RGB-D camera to track body joints and calculate ergonomic positions for handovers. Augmented reality is integrated to enhance collaboration from a human perspective. Machine learning techniques are combined with a Gaussian process model to predict hand position. Hyperparameter optimization and Artificial Neural Networks (ANNs) are used for time-series prediction due to their promising results. Model agnostic meta-learning is one of the most widely used meta-learning algorithms in human motion prediction research for an intuitive HRC. The authors propose integrating ML and GP regression for fast adaptation.

The study by Manns et al. [32] explored potential applications of Operator 4.0 within manufacturing systems. The Pupil Invisible, XSens MVN Awinda, and Manus Prime II are used in a technology setup to simulate human motion behavior in assembly tasks. These devices use inertial measurements to capture body motion data, while Manus Prime II incorporates inertial sensors into its finger-tracking hardware. The authors proposed a new method for identifying spatial region-based activity using real-time simulation, combining virtual environments with wearable full-body human motion capture, including eye tracking. The long short-term memory (LSTM) architecture detects lapses of attention, increasing safety and productivity in human–robot collaboration.

Papanagiotou et al. [34] reported the integration of gesture recognition and pose estimation in a professional environment, specifically an industrial assembly line, as part of Industry 4.0. The experiment involved assembling a TV panel using a robot, a GoPro Hero 9 camera, and an active vision system for pose estimation. RGB-D camera data were used for pose estimation, and a real-time DL module was used for gesture recognition. 3DCNNs and OpenPose were used for egocentric and operator skeleton extraction. Transfer learning improved gesture recognition accuracy by 11% when new users were introduced, and it increased to 98.5% after early termination.

Al-Amin et al. [35] presented a novel approach using two wearable devices, Myo armbands, to collect IMU data from assembly workers’ hands during tasks. The data are used to train two CNN models with identical architectures, which are designed to recognize both right-hand and left-hand actions. The classification outcomes are combined to generate a final action recognition result considering common collaboration. Transfer learning is used to adapt recognition models to new subjects not included in the training dataset. The study’s findings show that this approach significantly enhances prediction accuracy at both action and subject levels.

Choi et al. [36] provided a valuable use of Azure Kinect depth sensors to create a virtual environment for human–robot collaboration. Sensors scan the operational environment, creating a 3D point cloud of a virtual human–robot collaboration environment. Azure Kinect depth sensors capture RGB-D and skeletal data, merging them into a virtual environment through 3D tracking and matching. These data feed a Mask R-CNN, which detects and segments target objects, provides task-based guidance, and enhances synchronization between the physical robot and its digital counterpart.

In the field of human–robot control (HRC), a novel approach to natural human–robot control has been developed by Lima et al. [38] using a single depth-based camera, the Microsoft Kinect V2. The Kinect V2 is a depth-sensing camera that captures RGB-D data from users, enabling real-time hand-state classification. Positioned 0.9 m away, it uses pose mapping based on Thin-Plate Splines (TPSs) and Long-Term Recurrent Convolutional Networks (LRCNs), providing an intuitive user interface for teleoperation and improving accuracy in critical regions of the workspace. Cross-validation experiments show a higher accuracy of LRCNs compared to CNN classifiers.

The study presented by Mendes [39] developed a two-step hand gesture recognition algorithm using sEMG devices, specifically the Myo armband. The prototype device uses sEMG and IMU technology and a two-step gesture recognition algorithm. The first stage is segmentation, distinguishing between gesture signals and non-signals. Two algorithms, kNN and threshold-based, are tested. The threshold-based algorithm, using deep learning, outperforms the kNN algorithm, achieving 97% accuracy (despite kNN’s 92%) and enhancing human–robot collaboration in various settings.

Orsag et al. [40] investigated the development of a safe, flexible human–robot collaboration system using machine learning techniques like LSTM networks. The system uses the inHARD dataset, capturing 4804 action samples across 28 videos, and RGB data from three perspectives. The skeleton modality uses wearable IMUs for motion data. The method achieved 91.365% accuracy. The LSTM networks can achieve an accuracy of 91.365% on the training set, ensuring safe and efficient collaboration between robots and human workers.

A consideration that emerges at this point, and further details will be given in the discussion of the results, is that RGB/RGB-D and IMUs are solutions easily preferred over others in the data collection in these domains. Furthermore, the use of DL, particularly convolution-based architectures, seems at this stage to confirm the trend suggested by the productivity improvement studies. Additionally, tools like OpenPose are gaining importance also in this type of research when dealing with RGB data.

#### 3.1.3. Safety Support

In the context of the human-centered production system, the development of safe workplaces is of utmost importance. From this viewpoint, the study of workers’ well-being and efficiency of operations are channeled into ergonomics and occupational safety. The most adopted indexes include Rapid Entire Body Assessment (REBA), Rapid Upper Limb Assessment (RULA), Ergonomic Assessment Worksheet (EAWS), Ovako Working Posture Analysis System (OWAS) and Occupational Repetitive Actions (OCRA).

Taken together, the methods mentioned above act as architects of change within organizations, instilling a culture deeply committed to safety and well-being. They form the foundation upon which the edifice of safer, healthier working environments is built, reconciling employee well-being with operational efficiency while maintaining an unwavering commitment to formality. Following this perspective, other authors also consider ergonomics beyond typical ergonomic standard indexes. Along this, it is to be said that the traditional approach to the computation of risk based on the mentioned indexes relies on the evaluation given by an expert and on thresholds; thus, situations of uncertainty and subjectivity may arise [48,58]. This aspect pushes the research toward the exploration of novel approaches to risk assessment and safety support that promise to be less biased.

From this perspective, the work by Grandi et al. [45] showed a structured methodology for facilitating automatic data extraction from virtual analyses using digital production tools. Siemens’ EAWS-JACK worksheet automates manufacturing ergonomics assessment using a Siemens JACK 7.0 software toolkit. It accurately replicates operator postures and movements, allowing precise measurements of joint angles and body dimensions.

Maurice et al. [46] introduced a prototype e-glove equipped with three flexion sensors and four pressure sensors to measure general posture during industrial activities. EAWS is an ergonomics analysis tool used to improve working conditions in the industrial sector. Researchers used wearable sensors, optical motion capture technology, cameras, and finger pressure force data to train recognition models based on Human Movement Models. The dataset contains over five hours of data, including whole-body kinematics, finger force data, video recordings, and annotations from three independent human annotators.

Additionally, the need for continuous monitoring of the risk emerges in industrial scenarios. The study by Lorenzini et al. [58] presented an innovative methodology to continuously monitor and assess workers’ exposure to factors that cause work-related musculoskeletal disorders and to provide real-time estimates of physical stress in the workplace. The system uses an online multi-index human ergonomics assessment system, including the Xsens MVN Biomech suit, the Kistler Force Plate and the Delsys Trigno Wireless platform. As a comparison, the EAWS is used to provide a unique score, calculated by an expert, taking into account the entire work activity. The study used simplified activities and laboratory settings, and the EAWS score was calculated for each trial of each task. The authors concluded that the EAWS is a complex approach that can only be applied offline and is prone to expert subjectivity.

Nunes et al. [59] employed a comprehensive framework for monitoring human motion in industrial contexts and assessing work-related posture risks. The study focuses on a comprehensive framework for monitoring human motion in industrial settings and assessing posture risks. It analyzes joint angles using data-driven synchronization and kinematic descriptions. The framework compares joint angles with validated inertial motion capture systems. The study also explores an automated risk assessment tool (EAWS) for estimating risk exposure based on posture, strength, force, and repetition factors. The total risk score is calculated by summing the partial risk scores for all postures.

The work by Massiris Fernández et al. [48] introduced an innovative approach to ergonomic risk assessment using computer vision and machine learning techniques. The authors propose a method for ergonomic risk assessment and RULA scoring using digital video footage, allowing the cost-effective simultaneous handling of multiple workers. They use RGB cameras and OpenPose to detect joints and limbs in real time, identifying worker skeletons using open-source neural networks. The method is evaluated using real-world image datasets and outdoor work environments. Joint angle thresholds are set based on experiments, and size and confidence thresholds filter out spurious data. Front and rear camera views are recommended for optimal skeletal data quality. The authors occasionally infer joint positions in scenarios with occluded body parts, allowing RULA scores to be calculated in multiple video sequences.

As remarked previously, the role of artificial intelligence is not negligible in the context of physical ergonomics. Dimitropoulos et al. [50] investigated the use of artificial intelligence and wearable devices to improve human–robot collaboration during assembly tasks. The methodology focuses on enhancing collaboration between three modules: the Action Perception Module (APM), the Ergonomics Improvement Module (EIM), and the Learning and Programming Module (LPM). These modules monitor working conditions and adapt robot behavior to make it more human-centered. Data are collected using Kinect Azure sensors and a simulated shop floor digital representation environment. Communication is facilitated using a ROS-based architecture. The LPM module transitions from predefined program execution to automated, continuous learning and adaptation of robot motion. The Ergonomics Improvement Module (EIM) aims to reduce the physical strain experienced by operators working with robots.

SPECTRE is a deep learning network designed by Ciccarelli et al. [53] to classify workplace postures and assess ergonomic risks with minimal disruption. It uses 18 Xsens MTw (Wireless Motion Tracker) units for comprehensive whole-body monitoring, focusing on the upper arm, forearm, wrist, neck and trunk. SPECTRE uses a sensor-independent convolutional network-based learning model, Mediapipe, to identify and categorize workplace postures. The architecture includes a segmentation layer and a parallel convolutional layer that uses pattern recognition to assess postures and identify ergonomic hazards.

Guo et al. [55] proposed an integrated VR-based approach to improve ergonomic design in manual assembly and maintenance scenarios. The methodology involves the integration of VR hardware components, motion capture data, and an evaluation framework within the DELMIA environment to analyze ergonomic elements in a virtual industrial maintenance and assembly scene. The SHELL model is used, and real-time motion data are captured through immersive simulations. Tools include 8 infrared cameras and 41 optical markers. The DELMIA environment optimizes design evaluation and decision making, potentially outperforming desktop-based ergonomic assessment methods.

Panariello et al. [60] conducted a biomechanical analysis of the human movements and internal load dynamics of operators performing overhead drilling tasks. The study used infrared digital cameras, integrated force platforms and eight EMG sensors to analyze joint angles, torques and muscle activation. The study revealed a rise in shoulder torque, anterior deltoid activation, and biceps brachii activity at higher working heights, suggesting the potential for assistive devices like robotic exoskeletons to enhance task performance and worker well-being.

In light of continuous monitoring, Vianello et al. [62] proposed a comprehensive suite of tools, including the Xsens MVN motion-tracking suit, which has been developed to provide real-time ergonomic feedback to human workers during tasks, including physical interaction with robots. The Digital Human Model (DHM) visually represents specific areas and joints of the human body based on ergonomic metrics such as RULA. The suit incorporates, additionally to accelerometers, magnetometers and barometers to improve accuracy. Variational Auto-Encoders (VAE) generate Latent Ergonomics Maps (LEMs) to visually distinguish between non-ergonomic and ergonomic postures, enhancing the accuracy of the DHM.

The Operator 4.0 framework explored by Peruzzini et al. [49] focuses on human-centered principles and their applicability in computerized industrial environments. It aims to ensure operator safety by monitoring their well-being and workload while improving the process performance and overall quality of operations. Key components of the facility include an eye-tracking system (Glasses 2 by Tobii), a wearable sensor for the real-time monitoring of vital signs (BioHarness 3.0 by Zephyr), a video camera (GoPro Hero3) and digital human modeling software (Tecnomatix Jack, Siemens, 2017) for creating virtual environments and digital twins of monitored operators. The Vicon Optical Tracking System allows full-body tracking of operator positions and digitization of movements, enabling the creation of virtual factory prototypes by merging digital mock-ups with real operator manikins.

A web platform system has been developed by Generosi et al. [54] to facilitate a future human-centered factory by semi-automatically calculating multiple risk indices and providing advanced analytics to proactively improve the monitoring of ergonomic risks. The system, based on RULA and OCRA, aims to improve ergonomic risk assessment and safety in industrial environments by using deep learning models and video analysis algorithms to collect data on worker postures, grip types and body segment angles. Although this system offers a user-friendly interface for defining work cycles and tasks, it has limitations in hand detection and gesture recognition, particularly for gloved hands.

Lin et al. [57] aimed to develop a system that uses joint angle data from image-based motion capture technology to identify high-risk postures and prevent occupational injuries. The system uses RGB cameras like GoPro HERO 6 Black and Vicon, calculates risk scores using OpenPose technology, and uses a decision tree to determine the most appropriate assessment method. The system provides an automated and comprehensive means of assessing and preventing injury, identifying frames with high-risk scores for further improvement by ergonomists.

Paudel et al. [61] introduced a framework for automating the analysis of industrial worker poses to reduce the risk of long-term musculoskeletal disorders (MSDs). It presents a novel method for analyzing industrial worker pose using Yolov3.3. The framework uses 2D images and Darknet-53 video datasets to detect workers and determine their body regions. The Pose Net network estimates human joints, and the Body Angle Reliability Decision (BARD) network checks reliability. If satisfactory, the system converts 2D poses to 3D using the 3DMPPE pose net method. The framework automates ergonomic risk analysis using key features and scoring methods. The study yielded significant results, showing high accuracy in ergonomic scoring for well-executed postures and obstructed or occluded postures.

Automated or semi-automated MSD risk assessment poses challenges not only in the calculation of appropriate indices and scores but also in the suitability of image capture systems. In this light, Alvarez et al. [41] investigated the importance of measuring upper limb joint angles in occupational health, focusing on the limitations of traditional motion capture systems. It identifies tasks that pose a risk of workplace injury due to repetitive or awkward postures and uses inertial sensors such as accelerometers, gyroscopes and magnetic sensors to measure joint angles (XSens MTx sensors). The data are then analyzed to identify potential risks and areas for improvement. The findings form the basis for targeted interventions to reduce workplace injuries and improve overall occupational health.

Fletcher et al. [42] used inertial-recording-body technology to analyze ergonomic risks in manufacturing. The IGS-Bio v1.8 full-body suit employed in this study automatically documents body segments and joints, ensuring objective data collection even in obscured areas. The technology assesses musculoskeletal risks of different postures based on REBA criteria with three primary assessment stages and result tables divided into groups. The final scores assign comprehensive scores to specific postures, determining the urgency of remedial action.

One aspect that makes these studies of great interest is that ergonomic concerns can lead to changes in working posture or redesign of the workplace to meet safety standards. The 3DSSPP software (version 6.0.6) was used in a study by Golabchi et al. [43] to improve construction projects by increasing productivity, safety, quality and cost efficiency. An RGB camera collected data on site conditions, work tasks, and worker movements. Action recognition modules identified operations, creating a simulation model. A path-planning algorithm assessed biomechanical conditions, updating work and workstation design.

In the research led by Nath et al. [44], the authors aimed to automate ergonomic risk monitoring using body-mounted sensors and ML techniques. The framework aimed to reduce the duration of ergonomic risks. Data were collected using body-mounted smartphones, and HAR techniques were used to categorize workers’ activities (lift/lower/carry, push/pull or no-risk activities) with SVM classifiers achieving 80% accuracy.

A similar approach was adopted in the study conducted by Conforti et al. [47], in which 26 people were equipped with wireless IMUs to assess postural patterns during manual material handling tasks. The data collected were processed using eight wireless IMUs (MIMUs MTw, Xsens Technologies), and they were used to train machine learning algorithms to identify postural patterns associated with high biomechanical risk. The study utilized an SVM classifier with 92% accuracy to develop an experimental protocol for assessing postural patterns, identifying appropriate postures, and evaluating motion analysis using a wearable system and biomechanical model.

Mazhar et al. [51] suggested a framework for static and dynamic gesture recognition using simple RGB vision, focusing on spatial attention driven by posture; the aim of the study was to provide a vision-oriented approach to human–robot or human–computer interaction in both social and industrial environments, ensuring safety while managing robot actions. The framework, StaDNet, works solely with RGB vision, estimating, with OpenPose, the depth of operators and identifying the region of interest that includes their hands. The framework can be implemented on any RGB camera, and it provides a frontal perspective of the upper body and can integrate alternative pose extraction systems for specific application scenarios.

As illustrated by Mudiyanselage et al. [52], the use of surface electromyography (sEMG)-based systems and machine learning algorithms can be used to automatically detect harmful body movements during manual material handling tasks. The National Institute for Occupational Safety and Health (NIOSH) has developed a system using sEMG-based systems and machine learning algorithms to detect harmful body movements during manual material handling tasks. The system uses the lifting equation to determine safe weight limits and assess risk levels. The study focuses on everyday static lifting tasks and presents an innovative ergonomic workstation design using motion capture hardware and virtual reality.

A pragmatic approach to estimating joint stiffness was introduced by Phan et al. [29] in a tooling task. The method uses a customized handle with multi-axis force/torque sensors and sEMG technology to capture complex 3D dynamics of forearm and wrist muscle activity. Optical motion tracking is used to monitor the tool’s pose. Tested on an industrial robot and human operator, it shows a positive correlation between impedance values and forearm muscle activity. Infrared markers track tool position and orientation during polishing.

**Figure 2:**
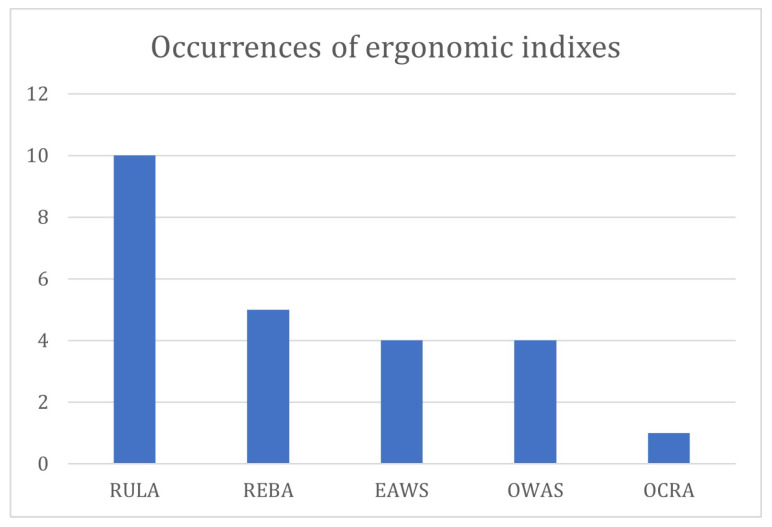
Number of occurrences of ergonomic physical indexes.

For the sake of clarity, Figure 2 provides the occurrences of the physical ergonomic assessment tools taken into consideration by the resulting articles.

The REBA is taken into account in the studies conducted by Fletcher et al. [42], Peruzzini et al. [49], Lin et al. [57], and Paudel et al. [61].

The studies conducted by Massiris Fernández et al. [48], Peruzzini et al. [49], Dimitropoulos et al. [50], Generosi et al. [54], Panariello et al. [60], Vianello et al. [62], Ciccarelli et al. [53], Lin et al. [57], Paudel et al. [61] and Guo et al. [55] incorporate the RULA ergonomic index. Vianello et al. [62] also introduce RULA-C because RULA’s time evolution often exhibits discontinuities and plateaus, making it less suitable for motion optimization and continuous postural assessment.

The ergonomic index EAWS is included in the studies led by Grandi et al. [45], Maurice et al. [46], Nunes et al. [59] and Lorenzini et al. [58].

The OWAS evaluation is factored into the research conducted by Peruzzini et al. [49], Lin et al. [57], and Paudel et al. [61].

The OCRA ergonomic index is a component of the research conducted by Generosi et al. [54].

### 3.2. Cognitive Ergonomics

In addition to physical well-being, the design of human-centered workplaces cannot neglect the emotional and cognitive aspect, as evidenced by the increasing number of studies on this aspect in manufacturing.

Cognitive ergonomics becomes core also in the development of human–machine interfaces and supervisory systems (SCADAs). In fact, these user interfaces should be taken into account in the context of workplace design in Industry 5.0, as the user experience (UX) plays a key role in the development of effective, human-centered solutions for human–machine interaction [86,87,88].

Nevertheless, cognitive workload and work-related stress are topics that have yet to be fully explored. In recent years, there has been a proliferation of studies seeking the best physiological parameters to characterize and detect these emotional states [89,90], but most of these have been carried out in the laboratory under controlled conditions. In industry and production, additional conditions must be taken into account, such as the tolerability of measuring devices by the worker and the adaptability of laboratory measurements to the production environment and to continuous monitoring. Mattsson et al. [63] proposed an industrial experimental setup to investigate how physiological measurement can be used to assess real-time operators’ well-being, performances and associated risks in an industrial context. They pinpointed heart rate variability (HRV), electrodermal activity (EDA), and respiratory factors as pertinent physiological indicators for monitoring changes in operator emotions and motivation. HRV data were collected using the Smartband 2 from Sony^®^ and the Activity bracelet E4 by Empatica^®^. Significantly, 50% of participants identified HRV as the most reliable parameter.

Nardolillo et al. [64] studied the HRV during simulated assembly line tasks to try to deduce the pattern of fatigue in different aged workers. Their findings show differences in HR between different body positions and genders and changes in the time domain with the increasing task complexity. Following the results obtained in this study, the HRV analysis gives effective cues to improve task demand decisions. Moreover, insights on the need for particular attention to the age of the workers in designing workplaces are given by the statistical analysis.

The appropriateness of task allocation in construction activities is the field of the investigation of Chen et al. [65]. They proposed an electroencephalography (EEG)-based approach to measure the mental load associated with construction tasks through the study of power spectral density (PSD) of EEG frequency bands acquired with a prototype EEG helmet. Even if preliminary, the statistical analysis conducted on PSD shows significant effects of tasks and frequency bands (particularly gamma band) on PSD and thus on mental workload. Their EEG results were validated by the ground truth NASA-TLX score.

Hwang et al. [67] employed a 2D emotional model, i.e., a valence–arousal emotional space, to quantify the emotional state of workers involved in construction tasks. A wearable EEG sensor was used to monitor the workers during construction activities. The results show that EEG-based affective indicators, particularly valence, are crucial in depicting the emotional state, even in the specific working scenario. Correlations with the levels of cortisol were computed to validate the results.

Bommer and Fendley [68] proposed a six-step theoretical framework for the analysis of mental workload in repetitive tasks in manufacturing activities based on cognitive ergonomics concepts and Multiple Resource Theory. Subjective measures provided by NASA-TLX and Workload Profile are adopted along with eye-tracking technology. Validation of the framework is performed with computer simulations, mathematical modelling and mental workload measures.

D’Addona et al. [69] stressed the need for an aware inclusion of the human factor in the increasingly automated and digital production systems. Their proposal for a human-in-the-loop factory adaptive automation framework was applied to two case studies, air traffic control and white goods production. The authors selected EEG as the physiological parameter to measure the operators’ mental workload.

The monitoring and assessment of mental workload in the workplace has the aim of giving crucial information on how to redesign the workplace to reduce workers’ stress and assist workers in stressing situations. In this sense, the study conducted by Landi et al. [70] presents a method for analyzing the mental workload of operators and adjusting assistive technologies accordingly, using affective robotics, to be applied in industrial settings. The operator’s stress level is monitored through heart rate variability (HRV), which is measured using a Samsung Gear S smartwatch equipped with a heart rate sensor. A group of 15 users teleoperated an industrial robot to perform a pick and place task. The operators’ R-R series, which is the time elapsed between two successive R waves, was monitored. When an excessive increase in the operators’ mental stress was detected, an assistive system based on virtual fixtures was activated to decrease their mental workload. The study’s findings indicate that the use of virtual fixtures in assistive systems can effectively reduce stress levels for users when the interaction task exceeds their sustainable mental workload.

As delineated by the previously mentioned works, EEG and HR are physiological parameters that well describe mental stress and workload. Along these, other measurements can be found in the literature such as electrodermal activity (EDA) and electro-oculogram (EOG). Kosch et al. [71] investigated the potentiality of EDA as a tool to real-time monitor the mental workload induced by two different assistive systems in manual assembly. This preliminary study suggests that EDA is a valuable measure of the cognitive load in this particular scenario along with working performance. As in many of the analyzed studies, the NASA-TLX subjective assessment was adopted, and data were analyzed by means of a statistical approach (ANOVA).

Papetti et al. [75] addressed their research to the human-centered connected factories; particularly, a real-world scenario is considered to suggest solutions to analyze the physical, cognitive and environmental ergonomics of industry workers. A number of different physiological parameters are monitored: HR, HRV, respiration, pupil size, and eye blink. Particularly, the authors remarked on the potentiality of EOG, suggesting its use in future studies in industrial settings. Contrarily, from the study by Van Acker et al. [76], it emerges that although employed eye-tracking glasses are felt as physically and mentally comfortable by participants in the study, pupil size does not provide significant information on the increased mental workload perceived by participants between different difficulty levels of manual assembly. This result, in contrast with laboratory studies, suggests that further advancement of this technology is needed for its use in real-world mental workload measures.

Albeit less frequently than in the case of the physical ergonomics discussed above, the use of artificial intelligence techniques, especially traditional machine learning but also neural networks, is also gaining ground in the analysis of cognitive ergonomics. Arpaia et al. [72] aimed to classify the stress levels of workers based on real-time EEG measurements acquired with single-channel EEG-SMT Olimex and Raspberry Pi^®^ 3. Other metrological references were used: standardized stress tests, observations from experts, and performance measures. The authors analyze the frontal asymmetry, which previous studies have shown to be a reliable EEG feature to characterize stress. Differently from the majority of the studies in the field, the authors adopted a machine learning approach to data analysis: SVM, k-Nearest-Neighbor (kNN), RF, and artificial neural network (ANN) were applied.

Bettoni et al. [73] used machine learning to propose a paradigm for adaptive human–machine collaboration to improve the productivity and well-being of workers. An injection molding manufacturing line involving humans and cobots is considered to test their proposal. Three wearable devices (Polar H10 chest strap, Empatica^®^ E4 wristband and Huawei Watch 2) were used to monitor HR, EDA, and skin temperature. The results show that the introduction of a smart decision-maker and a system of monitoring physiological parameters determines a reduction in workers’ mental workload. EEG signal acquisition and machine learning data processing is the approach proposed by Cheema et al. [66] to study the mental workload typical of multitasking activity, such as human–machine interactions. Their objective is to propose an automatic mental workload estimation to reduce the biases that can derive from evaluators and subjective analysis. A reduced set of features is used, and EEG channel selection is performed through mutual information. To classify mental workload, different classifiers are tested, and random forest (RF) shows the highest accuracy; the results support the hypothesis that EEG-based workload estimation via machine learning is a better solution compared to traditional subjective approaches.

Another issue to be stressed is that in the context of a smart, connected industry, the introduction of collaborative robotics to alleviate the workload of operators may lead, on the contrary, to increased mental workload [91]. For this reason, mental workload assessment becomes a useful tool for developing guidelines for mutual adaptation between humans and cobots through (1) human awareness and education in the interaction with the machine and (2) affective computing solutions integrated into collaborative robotics to calibrate the response of cobots to humans, thus approaching a truly human-centered collaboration [92]. In this light, Chen et al. [93] analyzed the effects of mobile robots on the mental workload of workers in smart warehouses. Order picking and assembly tasks are considered in three configurations of human–robot collaborations. The mental workload is assessed via pupil diameter and NASA-TLX score. An expansion of pupil diameter of workers emerges, and statistical data analysis (ANOVA) indicates an increase in mental workload due to collaboration with the mobile robot. This result is confirmed by the subjective assessment given by the NASA-TLX.

In the framework of information theory, Digiesi et al. [77] proposed an analytical model to measure the cognitive and mental workload of operators in smart factories via subjective (NASA-TLX) and physiological (HRV) parameters. ECG monitoring technology was used to monitor the cardiac activity of the operators performing standard tasks following the n-back procedure. The results obtained from the ECG monitoring are coherent with those in the literature; in fact, tasks with higher cognitive workload lead to an increase in cardiac activity. The same result was obtained in the experimental study from the subjective assessment. The Wilcoxon signed-rank test and the t-test are applied to analyze the data of the two assessments, HRV and NASA-TLX scores, respectively. Hopko et al. [79] studied the effect of different levels of automation in HRC on performance and on workers; sex, fatigue awareness and perceived workload and their relation with physiological parameters were considered.

Confirming its widespread use in stress studies, HRV is used with self-reports to monitor 36 workers on an electronics assembly line in the study by Khairai et al. [78]. Their objective is to measure the effect of workplace stress on workers’ HRV (monitored with EmWavePro^®^ system equipment) and to evaluate these effects compared to self-reports. Workers are divided into a treatment group and a control group; Mann–Whitney statistics is used to assess the significant difference between the two groups. The authors conclude that self-assessment and HRV-based evaluation of workplace stress are parallel, since subjects understand and report comprehensively their physical conditions. Moreover, the authors stated that HRV biofeedback interventions may help alleviate emotional symptoms in high-stress individuals.

The work by Argyle et al. [80] analyzed the relationship between task demand, fatigue, cognitive load and physiological parameters in operators involved in visual inspection tasks. FNirs, cardiac activity, respiration, and facial temperature were considered.

As observed so far, most of the studies on the well-being of workers tend to privilege one or the other aspect of well-being (physical or cognitive), moving away from the concept of holistic well-being cited above. A more complete analysis is proposed by Brunzini et al. [81], who presented an experimental study for the ergonomic evaluation of the physical and cognitive workload of operators to support the design of product and processes. Motion capture trackers allow analyzing of posture and movements, whilst eye tracking and bracelets play a role in monitoring the physiological response. The reliability of the monitoring system was supported by the use of traditional self-reports (NAS, Numerical Analogue Scale, and NASA-TLX). A video analysis enabled the performance analysis at the time of execution of the activities. A real industrial case was reproduced through a laboratory mock-up. Their results suggested that a whole analysis of the working conditions and of the human–machine interaction would be needed to perform a robust analysis enabling the optimization of the human and industrial performances. The authors suggested that a larger sample of participants would be needed for statistical validation of the proposed approach, and other devices should be tested to monitor the physiological parameters.

A slightly different aspect is analyzed by Lee et al. [74], who proposed an observational study on workers performing common construction tasks. The authors studied burnout in unskilled construction workers and proposed a protocol to include wearable sensors in burnout assessment. As previous studies reported, surveys and physiological parameters (HR) are involved; both physical and mental ergonomics are considered. Statistical analysis is performed using Partial Least Squares Structural Equation Modeling (PLS-SEM).

The influence on the mental load of the tasks’ complexity and assistance systems in manual mixed assembly is investigated in the work by Bläsing and Bornewasser [82]. The authors proposed a simulation of a real assembly task with two levels of difficulty and three different assistance systems; physiological responses and performance indicators were used to measure the mental workload. The results obtained show that the complexity of the assigned task has an influence on the mental load of the operator. Moreover, differences emerge between the types of assistance systems. The authors’ conclusion is that the combined approach of physiological parameters and performance indicators is apt to assess the operators’ mental load in assembly tasks, but that additional research is needed.

A machine learning classification of the mental workload is the approach proposed by Lin and Lukodono [83]. Physiological data collected from wearable sensors are used to feed an RF algorithm to predict the perceived mental load of an operator in an HRC scenario. The NASA-TLX score is used to self-report the perceived mental load. The features selected among those computed from the collected parameters allow a classification accuracy of around 94%, showing the potentiality of this type of approach. Moreover, the duration of the data acquisition (120 min) is in line with the real-world HRC scenario, further suggesting that studies in this direction would be needed to contribute to the human–robot co-evolution concept.

Mach et al. [84] study the feasibility of HR and arm motion measurements collected with a smartwatch to assess the mental workload of an operator. From their laboratory study, it appears that HR can be successfully adopted to monitor mental workload, but the authors suggest that its applicability may be limited to the measurement of mentally demanding activities only with a low level of physical activity. This suggestion opens the way for further contemplation of the practicality of implementing physiological measures in an industrial setting. Although the physiological approach is effective in stress detection, the proposed works do not seem to provide a sufficiently thorough analysis of its applicability in the real-world manufacturing scenario.

## 4. Discussion

In this section, the Research Questions (RQs) identified in the Introduction have been answered. Once again, the discussion is divided into two groups: physical ergonomics and cognitive ergonomics. Works related to physical ergonomics showed a common path: extraction of the human body synthesis parameters, synthesis parameters tracking, activity analysis, and recommendations. The first two steps aim to identify the anatomical districts employed to assess human activity in terms of gesture and posture and formalize them so that they can be tracked. For instance, joints such as shoulders, elbows, wrists, hips, knees, and ankles can be mapped into key points to compute Euclidean distances and angles. Thus, RQ1 aims to focus on these two steps in order to identify the most used technologies for acquiring these data. The activity analysis results are performed mainly through machine learning (ML) and deep learning (DL) to manage significant data dimensionality and cardinality. RQ2 details which artificial intelligence (AI) approaches are currently used to process the data. The last step is represented by the recommendations provided to improve productivity, man–machine interaction (MMI), or safety, namely the purpose of the selected works, which highlight the aim of assessing the workers’ well-being, as formalized by the RQ3. Answers to these RQs are aimed at a more aware choice of tools, methods, parameters and approaches to develop increasingly effective solutions within the domain of holistic well-being enhancement.

**Figure 3:**
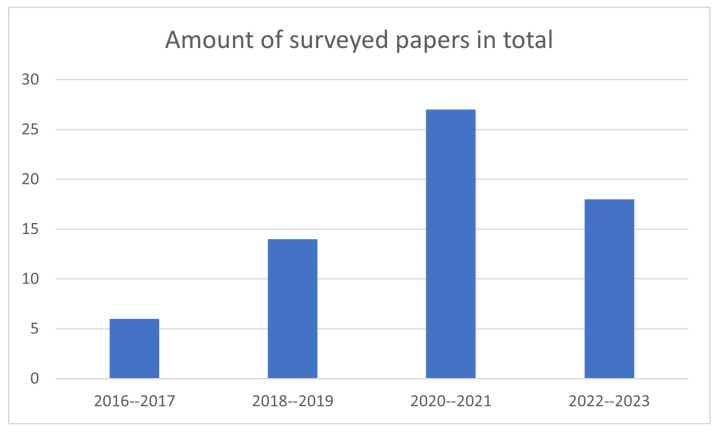
Amount of surveyed papers over years.

An increase in publications can be noticed over the years covered by the review, particularly during the 2020–2021 biennium, reflecting a considerable interest in the research on the topic (Figure 3).

### 4.1. Physical Ergonomics

Concerning RQ1, this literature review reveals that motion capture systems (MoCaps) in the industrial domain can be categorized into nine primary groups based on operational principles: wearable IMUs, RGB cameras, RGB-D cameras, optical IR marker-based systems, wearable sEMG sensors, force sensors, smartphone IMUs, multi-parametric wearable sensors for real-time vital parameters monitoring, and instrumented tool with force/torque sensors (Table A1 provided in Appendix A).

Optical technology is commonly considered top-tier, wherein body joint positions are estimated through triangulation using images from multiple cameras or data obtained from depth cameras. These developments have spurred research and innovation in RGB camera systems for 3D human movement recognition and RGB-D depth cameras that enable the measurement of an actor’s distance from the sensor by projecting an infrared light pattern and reading its reflection. These systems can make use of markers or operate without them. Inertial systems utilize accelerometers and gyroscopes to convey data about a performer’s movements and associate them with a virtual skeleton. This highly portable approach can be employed outdoors, although it may experience position drift. In particular, wearable sensors are suitable in environments where optical systems can be affected by occlusions or issues associated with reflective marker-based tools.

The ergonomic validation of a product or workstation is recommended whenever a human operator is responsible for manual tasks involving lifting, assuming awkward postures, or repetitive actions. Some indices like NIOSH, OWAS and lower-back analysis methods do not necessitate meticulous human body tracking; in fact, even a minor deviation in joint positioning (less than 20 mm) is generally inconsequential and does not influence the analysis results [85]. Therefore, while it might be simple to calculate skeletal joints using a Kinect camera, RGB can be employed to monitor extensive environments under diverse conditions. After proper data acquisition, software solutions like DELMIA, SIEMENS JACK, and others can compute ergonomic metrics from Mocap data. However, for a comprehensive physical ergonomics analysis, the worker’s gesture and posture recognition data with adequate accuracy and precision for transmitting data to any DHM should be provided. In this sense, certain technological limitations, among which low camera resolution, sensor invasiveness, and occlusions, still impede the adoption of MoCap and DHM solutions in the industry, since even a slight divergence from the actual position can impact the calculation of ergonomic indices. Furthermore, there are fewer challenges related to the misalignment of virtual body segments during intricate activities [26].

The literature analysis shows that the most adopted technology is represented by wearable inertial measurement units (IMUs), adopted in fifteen articles, followed by the RGB cameras (twelve articles), the RGB-D cameras (eight articles), the optical IR marker-based (seven articles), and the wearable surface electromyography (EMG), which was employed in five articles. Among the cited papers, eleven of them integrated different technologies; in particular, multi-modal integrated assessment systems are adopted in seven works.

**Figure 4:**
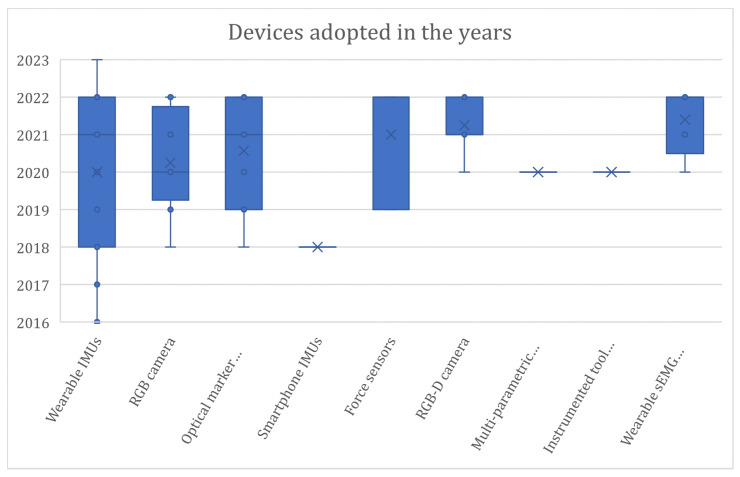
Adoption of the different devices in the time span covered by the review.

Figure 4 shows the distribution of the devices in the considered time span. Considering the time distribution of the technologies adopted over the years, IMUs are distributed over the entire time span, while cameras are most used from 2019 onwards. In particular, RGB-D cameras are concentrated from 2021 onwards. These data suggest that the technological development of RGB cameras, particularly RGB-D cameras, seems to push researchers to prefer this type of technology to wearable IMUs. Among the reasons behind this change in the data acquisition approach is the huge technological development of RGB-D cameras [94] that made it possible to improve the devices in terms of resolution, frame rate, and flexibility related to the operative conditions both for the color and the depth streams. A similar consideration can be made for surface electromyography (sEMG) wearable sensors; their adoption is concentrated from 2020 onwards. This relatively new technology can detect the electrical signals generated by muscle cells when they contract, preserving the naturalistic context of the acquisition thanks to being minimally invasive. It can be seen that seven out of eight of the articles using RGB-D concern applications of human–robot interaction and, thus, collaborative work; in fact, the additional spatial information can facilitate the monitoring of the pose and movements of the operator and the collaborative robot more than other types of cameras. Moreover, a slight preference toward RGB-D cameras is also registered in human–robot collaboration (HRC).

Analyses relating to human–robot interaction are core, since robots are increasingly required to achieve greater interaction and collaboration with human workers, surpassing mere coexistence. This requires real-time communication and coordination between the worker and the robot, which is achieved through on-site sensing, data processing, advanced computational infrastructure like cloud computing [30], and a user-friendly interface. In this scenario, gesture recognition becomes the core for implementing a naturalistic interaction. In this sense, the goal of gesture analysis is to extract unique features describing manual and finger movements regardless of the device used to record them. However, these characteristics regarding camera-based systems cannot be directly identified from video frames without considering the temporal correlation. The examination of video streams raises concerns that are distinct from image handling. Extracting necessary traits normally necessitates several preliminary stages not directly associated with the fundamental aim. This supplementary procedural component can create difficulties that may prove challenging to surmount in designing a solution for physical ergonomics assessment. Hence, it is pragmatic to evaluate the possibility of detecting human body language via hand gestures using pertinent gesture feature data [21]. However, several recent trends are increasing the demand for research in the field of human–machine interaction, of which the Industrial 5.0 revolution is a key driver. This revolution promotes the adoption of advanced digital technologies to facilitate human interaction with products and machines, thereby increasing productivity and reintegrating individuals into the modern workforce. The Industry 5.0 approach facilitates connectivity between different production resources, such as machines and workstations, enabling the generation and sharing of information to create self-adaptive, predictive, and automated decision-making processes. An empirical study [49] has demonstrated the effectiveness of this approach and the value of monitoring human physiological responses in defining socially sustainable workplaces. In this sense, virtual and mixed reality setups foster designers and engineers in their real-time design solution validation [49], making gesture recognition even more core. For instance, previous research has shown that the early prediction of human grasping movements can help the robot plan its trajectory while avoiding the area the human intends to occupy. This leads to smoother collaboration in the shared workspace.

Table A2, provided in Appendix A, shows the relationship between the employed acquisition devices usage and the monitored activity. Since there could be more than one purpose of use in a paper, as shown in Table 3 (e.g., “Pose estimation and Gesture Recognition”), these were considered separately in Table A2, in Appendix A, to develop further analyses. Force sensors, multi-parametric wearable sensors for real-time vital parameters monitoring, and instrumented tools with force/torque sensors are typically used in integrated systems as highlighted, for instance, in gesture recognition and estimation of human operator joint stiffness. This analysis shows that wearable IMUs and RGB-D cameras are used for monitoring the largest number of activities (six), followed by oMocap devices (three), proving once again the flexibility and efficacy of adopting such technologies. From the monitored activities perspective, gesture recognition and pose estimation were investigated using different devices eight and five times, respectively, to witness the primary importance of developing user-centered solutions. The other categories identify monitored activities often used as support for further evaluations. For instance, action recognition allows for a semantic comprehension of the workers’ activity, while human motion prediction aims to describe the dynamic behavior of the workers even away from their workstations or during position changes. Undoubtedly, this approach allows for a deeper analysis of the production process in terms of productivity and safety.

Regarding RQ2, the literature analysis clearly showed the wide diffusion of adopting an artificial intelligence (AI) approach for data processing. AI refers to the simulation of human intelligence in machines that are programmed to think and learn like humans. AI is typically implemented through machine learning (ML) and deep learning (DL) techniques, which involve the use of algorithms that allow systems to learn and improve from experience without being explicitly programmed. Thirty-two articles adopted these approaches singularly or combined as shown in Table A3 provided in Appendix A.

Machine learning analysis is often employed when sensor data are employed, particularly human activity recognition (HAR), offering the potential to consistently and objectively determine time data. This choice is fostered by the possibility of extracting features from the data, from which it is possible to identify and analyze patterns and trends for further classifications. For instance, in the work by Niemann et al. [33], the position of the trolley handle and the packing table were the most informative for activity recognition, properly identifying the correct features to perform the classification. There is no clear predominance of one algorithm over the others for what concerns ML; only a slight prevalence of SVM, HMM, and kNN emerges, confirming that ML techniques have strengths and weaknesses strongly dependent on the application domain and the available data in terms of dimensionality and cardinality [95]. To provide a clearer understanding of the technical aspects of one of the algorithms identified as being prevalent in the analysis, some technical aspects are provided. SVM has been demonstrated to be effective for the classification of specific activities or gestures particularly in the context of hand gesture analysis and human activity recognition in warehouse operations (Table 3). Its popularity can be attributed to its robustness in high-dimensional spaces and effectiveness with limited data [96]. The choice of the kernel (linear or radial basis function), the regularization parameter, and the gamma parameter are decisive for defining decision boundaries and ensuring the ability to generalize to new data [97].

On the other hand, deep learning is the most adopted technique, and within this group, Convolutional Neural Networks are the architectures used. CNN was found in 19 out of 42 papers relating to gesture and posture recognition with/without attention to physical ergonomics. Convolutional networks differ widely from other machine learning and deep learning algorithms in frequency of use, as the second most frequently used (i.e., SVM) is found four times. OpenPose and Mediapipe, based on CNN architectures, are considered CNNs within the Table A3’s categorization, which is provided in Appendix A. Nonetheless, although using a combination of CNNs to extract characteristic features and identify human body joints from RGB images, YOLO is considered separately, since it incorporates specific design elements to efficiently perform real-time object detection. The diffusion of OpenPose (eight articles) is related to the required input data. In fact, only RGB images are used to feed the neural network, resulting in the need to provide a simple RGB camera for monitoring the activity. The drawback of having a simpler solution is the impossibility of managing and including 3D data in the analysis.

From this perspective, ML requires fewer sensor data, reducing training time, memory usage, and overall computational costs [98], which was very effective in those scenarios where there are a lack of data or an unavailability of computational power. On the other hand, DL techniques offer several advantages, such as the possibility of learning from unlabeled raw sensor data, solving complexities in inter-class and intra-class variability, and avoiding data preparation and dimensionality reduction, but a large amount of data is mandatory to avoid overfitting and consequently reaching satisfactory performances. Enhancing deep learning models with a larger dataset (images, videos, sEMG signals) will improve existing machine learning models and allow for the evaluation of more advanced algorithms [52], eventually adopting multi-modal approaches.

An interesting viewpoint to frame the context of assessing the workers’ well-being is to consider the adoption of the algorithm throughout the selected period. Convolutional Neural Networks (CNNs) are the most adopted classification algorithms throughout the selected period, especially for tasks related to image and video analysis. The range of industrial contexts of CNN adoptions includes hand gesture analysis, pose estimation in collaborative workspaces, and human activity recognition during order-picking processes (Table 3). This capacity to capture spatial hierarchies in data is the source of their effectiveness. To optimize the performance of CNNs, researchers adjust the parameters of the network, including the number of layers and filters, to enhance the extraction and classification of features. The learning rates and optimization methods employed (e.g., Adam, stochastic gradient descent) are meticulously calibrated to guarantee convergence and avert overfitting [97]. In the context of deep learning algorithms, another notable architecture, before returning to highlight the importance of CNNs, is LSTM, which is particularly adept at modeling temporal dependencies, and it has a role of significance importance in the field of action recognition and human motion prediction, particularly in data-rich environments characterized by sequential data, such as manual assembly tasks on shop floors. LSTMs are preferred for their capacity to retain information over extended sequences, which makes them well suited for predicting future movements based on past actions [99]. The number of units, learning rate, and sequence length are important parameters for LSTMs, as they influence the network’s ability to capture temporal dynamics in human motion. Readdressing the previous topic, CNNs have gained immense popularity in the field of deep learning after having consistently outperformed traditional computer vision techniques in various image recognition competitions, including the ImageNet Large Scale Visual Recognition Challenge, but also for the availability of pre-trained models; in fact, transfer learning allows researchers and practitioners to leverage the knowledge learned from other large datasets and apply it to smaller, domain-specific datasets. This is especially valuable when working with limited labelled data. When analyzing the algorithm distribution excluding CNNs in order to focus on other solutions in the industrial domain, it can be seen that machine learning (ML) was widely adopted before 2021, occurring fifteen times over the six related to the adoption of the deep learning (DL) approach. By contrast, DL became more popular in the last few years, from 2022 onwards, occurring five times as well as ML, which still represents a valuable option in specific scenarios such as the paucity of the data for the training process. This trend further confirms the use of DL in current years, which is fostered by technological development; in fact, nowadays, simple neural networks can be trained and tested even in customer-grade computers and by the availability of external providers to exploit cloud computing, which is an innovative technology to outsource the required computational need to remote servers.

Considering RQ3, the works also provide an ergonomic assessment of worker well-being even if ergonomic assessment tools were employed in only 22 of the 42 considered articles. Musculoskeletal disorders (MSDs) are prevalent health issues affecting individuals across various professions, leading to long-term disability and economic consequences. These disorders encompass a range of conditions caused by strain on internal body parts during movement, including muscles, nerves, tendons, joints, cartilage, and spinal discs. Examples include carpal tunnel syndrome (CTS), tendonitis, and bursitis. Work-related musculoskeletal disorders (WMSD) are occupational injuries that can lead to permanent disabilities, affecting professional and daily life, and they are a major cause of sickness absence, disability, and productivity loss in developed countries. In the European Union, they constitute over 50% of occupational diseases and contribute to more than 40% of economic losses related to workplace health and safety issues. In the United States, they account for over 30% of non-fatal illnesses and injuries. An effective ergonomics program should incorporate evidence-based ergonomic risk assessments (ARs) to identify and rectify ergonomic issues in different work scenarios. Various methods and tools for ergonomic ARs, such as self-assessment, human observation, direct measurement, and computer-based assessment, have been developed. Real-time body posture monitoring, particularly in manufacturing industries, can provide valuable data for enhancing working conditions and logistics optimization. These computer vision-based technologies not only classify human activities but also assess posture and movement safety.

In this sense, safety was shown to be the most relevant purpose of ergonomics studies. Neglecting to address unsafe postures and movements can lead to injuries, harming an employee’s physical well-being, morale, quality of life, safety, and productivity. It is preferable to prevent these issues, and one effective way is by raising workers’ awareness of risky postures and movements. Recently, wearable sensing technologies have enabled the collection of near-real-time data for analyzing worker safety and health conditions. These technologies are cost-effective, user-friendly, highly accurate, and non-invasive, proving to be not an obstacle but rather a catalyst for productivity. Moreover, maintaining proper body posture, especially during physically demanding tasks like material handling, can reduce the risk of work-related musculoskeletal disorders (WMSDs), decrease absenteeism, and enhance work productivity and safety. Inadequate ergonomic conditions in work environments can result in severe work-related musculoskeletal disorders (WMSDs), potentially leading to significant disabilities. This combination of assessments opens new possibilities for ergonomic analysis, considering specific tasks that may vary by time and location [48].

The most adopted ergonomic index is RULA, applied in ten articles, which is followed by REBA and EAWS (four) and OWAS (three). Undoubtedly, upper limbs are the most investigated as well as the most involved anatomical districts in executing tasks in the industrial domain; indeed, ergonomic studies are performed with manifold purposes: increasing productivity by enhancing manual processes, improving man–machine interaction, and designing new solutions to support and guarantee safety working conditions to the operators. Among the other indices, OCRA is computed only in one work. In eight works, custom indexes are defined. Four articles involve more than one standard index, while RULA combines other ergonomics evaluations in one work. Non-standard indexes are evenly distributed over the period, while a massive use of RULA emerges from 2020 onwards (Table 4), which is possibly related to the ongoing diffusion of collaborative robots. Of the twenty-two articles on physical ergonomics that were analyzed, only five articles consider more than one ergonomic evaluation: in particular, four consider several standard ergonomic indices [49,54,57,61], while one combines a standard evaluation index with a non-standard evaluation [62].

Traditional kinematics-based ergonomic assessment tools, such as RULA, REBA, and OWAS, use human joint positions to calculate an ergonomic score for a given body posture. While kinematics-based scores are quick to calculate, they may need to fully consider the dynamic aspects of the task [62]. The introduction of wearable technology, such as motion data and physiological metrics like blood volume pulse (BVP), electrodermal activity (sweat), electrocardiogram (ECG), respiration, and electroencephalogram (EEG), is poised to revolutionize physically demanding sectors like construction and manufacturing, enabling the more accurate prediction of ergonomic risks and physical injuries [44]. Proposing a framework can serve as a tool to assess risks in industrial production lines, offering quantitative insights into the actual exposure of workstations and operators. This framework can also be used to support operator training and evaluate the effectiveness of ergonomic interventions. In the future, using a more complex upper body model could be a viable alternative to obtain a more accurate estimates of degrees of freedom (DoF) for extreme positions, helping to improve motion tracking [59].

The aforementioned considerations allow for affirming that the actual panorama proposes (1) various solutions based on the activity to be monitored; among these, (2) cost-effective solutions (e.g., RGB, RGB-D cameras) are proposed, which give satisfactory results and which (3) represent less invasive alternatives to wearable or marker-based devices. From the perspective of a multi-modal approach, this latter aspect takes on fundamental importance when (4) integrating these solutions with cognitive monitoring devices that must be worn by the worker in order to acquire physiological parameters (and can be therefore uncomfortable when combined with other wearable devices). Additionally, (5) the explored solutions propose a wide panorama of approaches typically based on AI for body/gesture recognition/tracking and posture monitoring/classification that are accessible to researchers from different backgrounds. (6) The combination of these technological solutions and the ergonomic risk assessment indices provides the potential for the development of automatic assessment systems. This, in turn, may result in the elimination of any potential biases that may be introduced by the evaluator’s experience. On the other hand, this aspect introduces a limitation of current approaches, in which (1) fully automated evaluation cannot replace that of an expert evaluator. In this regard, automated assessments should be developed to support the evaluator in identifying risks and to enhance performance, the quality of worker–machine interaction, and overall safety. Another limitation found in the current literature is the (2) scarcity of precise indications regarding the operational conditions in which each of the proposed solutions can be effectively adopted within the complexity of a real production system. For instance, if we were to adopt solutions that are low-cost and minimally physically invasive, such as RGB/RGB-D cameras, (3) considerations should be made regarding the limitations arising from the acquisition of sensitive data (e.g., the worker’s face), which have minimal impact at the level of scientific studies but become significant when transitioning from experimental settings to real-world workplace applications. In general, (4) the acceptability of monitoring in a real work context is likely lower than in a study setting. A meticulous assessment of the positioning of the cameras allows, where possible, not to capture the face of the operator; in this way, together with compliance with the specific regulations on sensitive data, a clear statement to the worker of the purpose, and an exhaustive explanation of the benefits derived from a conscious and focused application of well-being monitoring solutions, this limitation can at least partly be managed. The influence of this human factor on the actual accuracy of the proposed methodologies should be investigated in parallel with technological aspects in order to achieve the development and practical implementation of truly human-centric solutions that are accepted and embraced by the worker.

### 4.2. Cognitive Ergonomics

**Figure 5:**
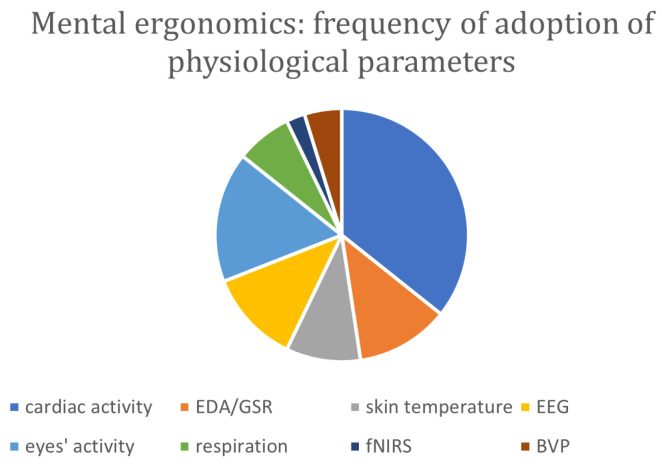
Graphical representation of the frequency of adoption of each of the physiological parameters.

From the analysis of the collected papers, it emerges that cardiac activity is the most used parameter (ECG, HRV, HR), which is followed by eye activity (eye tracking, eye blink, pupil size), electroencephalography (EEG) and skin conductance (EDA, GSR). In fewer studies, the use of breathing factors, blood volume pressure (BVP) and fNIRS emerges. The frequency of use of each considered parameter as emerged from the literature is represented graphically in Figure 5. The prevalence of cardiac activity also emerges from studies aimed at analyzing the cognitive load and stress in other applications different from industry, suggesting its efficacy and ease of acquisition.

**Figure 6:**
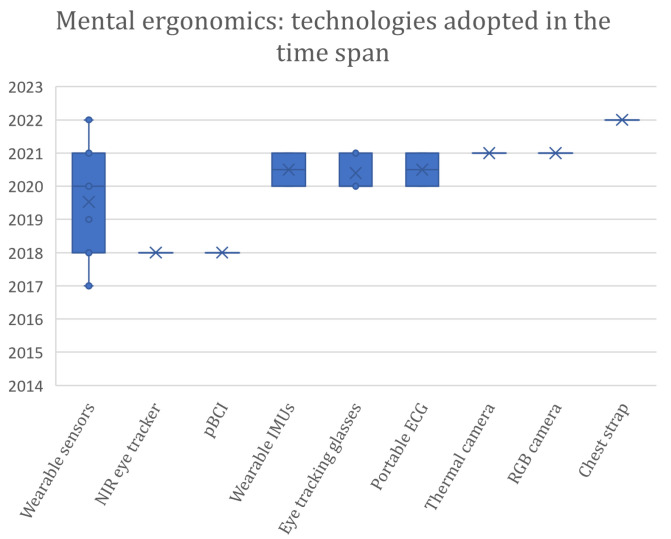
Adoption of the different technologies to acquire physiological data in the time span considered.

**Figure 7:**
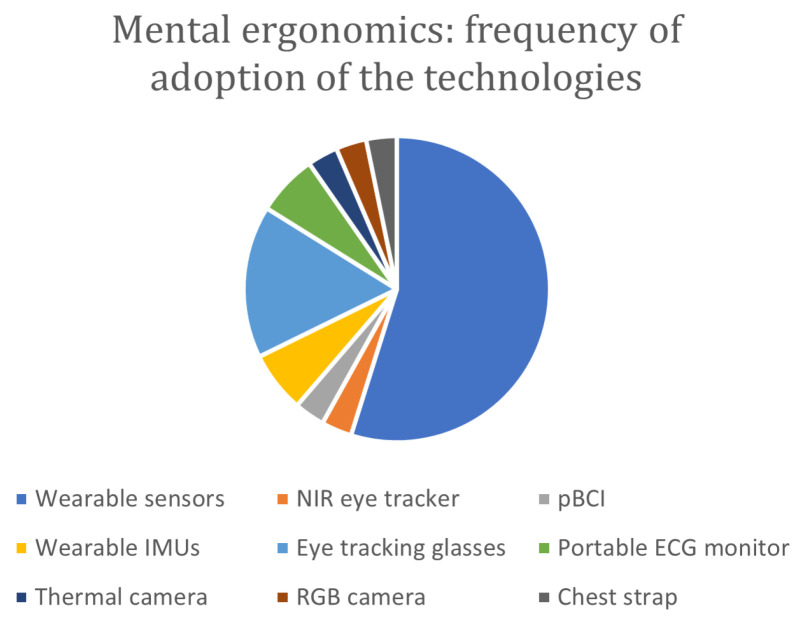
Graphical representation of the frequency of adoption of the data acquisition technologies.

Considering RQ1, the distribution of the technologies adopted in the considered time span is reported in Figure 6. The most widely adopted technology is represented by wearable sensors, which are suitable for acquiring data in a non-invasive and cost-effective manner without requiring too extensive training (Figure 7). On the other hand, a limitation of these devices, particularly concerning cardiac activity, is the fact that their applicability in the study of work stress and mental ergonomics in real working scenarios may be limited to activities with low physical effort [84].

**Figure 8:**
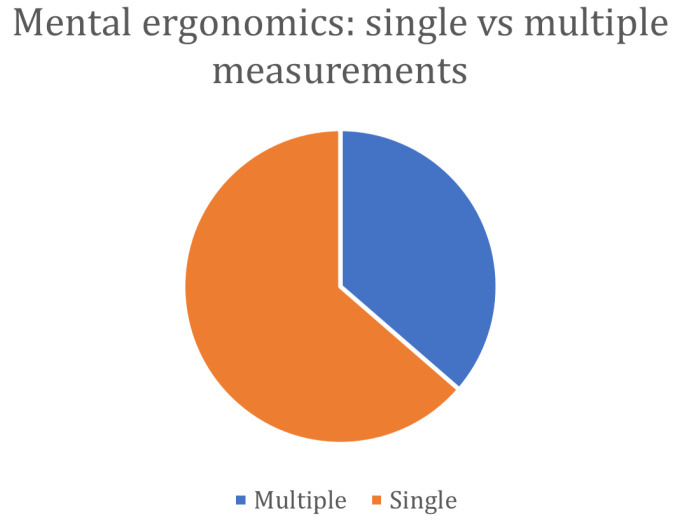
Graphical representation of the frequency of adoption of single vs. multiple physiological measurements.

The majority of the studies involve only one type of physiological parameter (Figure 8); moreover, the studies in which more than one parameter is recorded do not lead to the development of an effective multi-modal system of assessment of the cognitive load. From this viewpoint, the study of multi-modal systems could allow for obtaining more effective monitoring tools, where the application limitations of one parameter could be mitigated by the effectiveness of others [84,100,101]. In this sense, in addition to studying the most suitable physiological parameters in the industrial context, it would seem reasonable to study their most effective combination. On the other hand, a number of studies would be needed on the ergonomics of multi-modal systems in real-world industrial scenarios; indeed, although a multi-modal system could potentially be more comprehensive, the presence of multiple sensors could lead to a reduction in the physical and mental ergonomics of the operator. One further point that emerges is the paucity of studies in which the worker’s well-being in the industrial context is considered simultaneously from the physical and cognitive point of view [75,81]. The parallel study of these two aspects seems of fundamental importance from the perspective of Industry 5.0.

**Figure 9:**
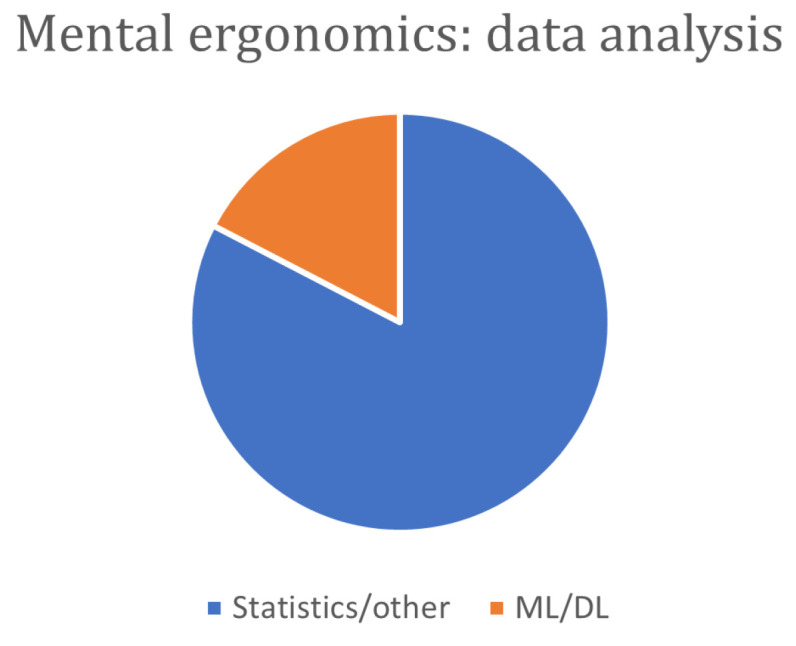
Graphical representation of the frequency of adoption of statistical vs. machine/deep learning approaches.

Concerning RQ2 and RQ3, the analyzed studies reveal a prevalence of the statistical approach to cognitive ergonomics data analysis; in contrast, few studies use machine and deep learning approaches (Figure 9). This suggests that research at present is primarily directed at the detection of a state of cognitive overload and stress with a view to determining the appropriateness of physiological measures and not yet at the construction of systems for monitoring, classifying, predicting and correcting the state of the operator, which would feature a greater involvement of AI.

A further consideration is that many studies in the literature deal with mental/cognitive load and stress, particularly in air traffic control and aviation [102,103], driving [65], software development [104], office work [105,106,107], and marine training [108,109], but there are few where the application is in industrial and production contexts. In the latter, they are mostly considered standard activities and not real industrial production applications. This reflection is closely linked to the lack of studies analyzing the actual applicability of the proposed physiological measures in real industrial scenarios. In fact, although the measures proposed have proven validity in stress analysis, there is a lack of detailed analysis of which of these measures, and to what extent, could actually be adopted in the industrial context in order to achieve real human-centered workplace design. Furthermore, a lack of studies emerges in which activities are carried out in industrial settings and integrated with VR/AR technologies; this type of study would seem to be indicated considering the growing interest in these technologies within Industry 5.0. Additionally, it would be helpful to have a deeper knowledge of the effects on the cognitive load induced by these technologies. In this sense, studies organized on different levels of difficulty of the tasks would be needed to discriminate those situations in which VR/AR technologies could cause a worsening of the state of the operator and those in which these technologies could determine an improvement or at least match more traditional approaches [110].

Finally, most of the analyzed studies do not seem to be conclusive; in fact, in several cases, further studies are addressed as necessary.

Based on these considerations, we can assert that (1) the literature on physiological parameters, which generally allow the study of stress and cognitive load, is extensive; less extensive is the literature investigating their application in industrial settings. In the latter, as can be seen from the analysis of the articles considered, (2) there are several parameters recognized as effective (HRV, EEG, EDA) and others whose effectiveness is not unanimously agreed upon (e.g., HOG). Additionally, (3) devices for acquiring such parameters are available at an affordable price, and (4) although they are wearable devices, they are not particularly invasive. Another aspect that makes these solutions attractive is (5) their truthfulness, which renders them more reliable than traditional questionnaires. This latter strength becomes a potential limitation when considering the implications related to (1) the sensitivity of the acquired data, and consequently, (2) the acceptability of a type of monitoring that the worker may perceive as invasive. A clear statement of the purpose of the acquisition of data from workers and compliance with specific regulations on sensitive data are fundamental to the application of cognitive monitoring. Furthermore, monitoring should be aimed at acquiring the necessary data to study ergonomic problems and should be discontinued when the problems are solved.

As with physical monitoring, (3) the literature analyzed highlights the paucity of studies evaluating the actual applicability of the proposed parameters in real industrial scenarios. For instance, EEG emerges as a powerful measure in detecting stress and cognitive load, but the acquisition devices could be poorly tolerated for prolonged periods, thereby proving unsuitable. Similarly, HRV due to stress can easily be masked by HRV due to prolonged or physically demanding work, as suggested by Mach et al. [84].

In addition to the suggestions this work aims to make based on previous literature, further suggestions can be made based on the experience of the authors [111,112,113]. Considering the EEG, two types of devices have been explored in the authors’ experience, namely a 14-channel saline headset (Emotiv Epoch X) and a 32-channel saline head cap (Emotiv Epoch Flex). Concerning the first, the reduced number of channels makes the positioning on the head of the subject easier, whilst the second provides greater granularity of the acquired data but requires more time and experience for the effective positioning.

Further issues can be due to the fact that in industrial applications, personal protective equipment (PPE) may be required. As an example, the headset is unsuitable when PPE such as a safety helmet is worn; this aspect can be mitigated with the use of the head cap, but both devices are not suitable when a PPE such as a coverall is worn for prolonged time, making it impossible to rehydrate the saline sensors. An additional problem is represented by the tolerability of the devices. In fact, if the flexibility of the head cap makes it tolerable even for a prolonged time, the headset can be tolerated for not more than 30 min. On the other hand, a more stable signal is obtained with the headset. Regardless of the type of device, problems were encountered in data acquisition when performing tasks in which the subject was required to move in space and to interact with real or virtual objects. Regarding the other measurements suggested (EDA and cardiac activity), the authors experienced the use of the Shimmer3 GSR+ device for GSR and photoplethysmogram (PPG). Concerning GSR finger sensors, issues can be related to the use of PPE such as gloves, particularly tight gloves, causing a deterioration of the signal. On the other hand, the optical pulse sensor for PPG can be placed on a finger or on the ear lobe; the latter position allows to overcome problems due to gloves and is compatible with PPE such as security helmets and coverall.

## 5. Conclusions

The integration of physical and cognitive monitoring methodologies in the industrial scenario is an ambitious goal, as many limitations and implications have to be addressed, but it appears necessary in order to realize an even more ambitious goal, namely the realisation of the human-centric factory invoked by the Industry 5.0 paradigm. The knowledge of the state-of-the-art methodologies for the physical and the cognitive monitoring is necessary for an effective integration of the two and for a realistic adoption in the real industrial environments. This study contributes significantly to the field by providing a comprehensive overview of the current state of the art in both physical and cognitive monitoring methodologies. It offers researchers and practitioners a clear path to bridge the gap between these two domains. The findings presented emphasize the feasibility of integrating these approaches and highlight their critical role in advancing the Industry 5.0 agenda.

The graph represented in Figure 3 illustrates an upward trajectory in the number of surveyed papers over time, indicating a growing scholarly interest in the subject. From 2016 to 2017, there was a consistent increase in the number of publications with a significant surge in the 2020 to 2021 period. This peak likely corresponds to an intensified focus on Industry 5.0 concepts, especially during the rapid evolution of the industry due to digital transformation and the need to improve worker well-being. The subsequent period (2022–2023) exhibited a slight decline, yet the number of publications remained considerable, indicating the research field’s continued relevance to academic and industrial communities.

From a physical ergonomics perspective, designing workspaces, tools, and equipment that prioritize human well-being, comfort, and safety is crucial. As Industry 4.0 introduces more collaborative robots and automation, it becomes imperative to ensure that the physical environment is optimized for seamless human–robot interaction, enhanced productivity and safe working conditions.

Simultaneously, cognitive ergonomics takes center stage in Industry 5.0, where complex decision making, problem solving, and human–machine interactions become integral components. Designing interfaces and systems that align with human cognitive processes, reduce cognitive load, and promote effective information processing are core.

Moreover, the integration of artificial intelligence and machine learning in the factory requires a careful consideration of how humans interact with and trust these technologies. In this sense, training programs and user interfaces must be designed to enhance cognitive compatibility and facilitate a smooth transition to advanced technologies. It is recommended that future research concentrate on the improvement of these algorithms with a view to a more effective integration of physical and cognitive data. This would facilitate the implementation of real-time feedback mechanisms that support dynamic and adaptive workplace environments.

From the technological perspective (RQ1), this literature review observed that wearable IMUs were predominantly employed to track the operator’s state, offering an alternative to occlusion issues. However, optical systems like RGB and RGB-D cameras offer more freedom and are less intrusive. Furthermore, depth cameras are more commonly used in human–robot collaboration scenarios, which may pose challenges in real-time communication. From the viewpoint of work stress and mental ergonomics assessment, cardiac activity is the most adopted physiological parameter, with wearable sensors being the most adopted technology for data collection and monitoring. However, these conclusions must be considered in relation to the type of activity performed by the operator, as measuring cardiac activity as an indicator of work-related stress loses validity in the presence of physical exertion. A multi-modal holistic approach could provide advantages, but further studies are needed to identify the best combination of parameters and quantify discomfort due to multiple wearable sensors. The technological insights provided by this review are useful for the guidance of future advancements in ergonomic monitoring. By identifying the strengths and limitations of current technologies, this study aims to contribute to the foundation for the development of more integrated and user-friendly solutions that can adapt to the specific needs of different industrial contexts.

Regarding RQ2, OpenPose and MediaPipe are popular deep learning algorithms used in physical monitoring data processing. CNN is the most commonly used algorithm, offering, as is well known, state-of-the-art performance, allowing for learning from raw sensor data without data preparation or dimensionality reduction. On the other hand, classical machine learning requires less sensor data, resulting in reduced training time, memory usage, and computational costs. Quite the opposite, statistical analysis is the most commonly used solution for analyzing cognitive monitoring data. This leaves the way open to the study of solutions in which the involvement of artificial intelligence can facilitate the integration of mental ergonomics into systems for assessing the overall state of the worker as an effective tool to support the development of the factory of the future, also considering the wide adoption of artificial intelligence in gestural and postural analysis. Further research could integrate data pertaining to both physical and cognitive aspects, thus creating more adaptive systems that are capable of responding in real time to the ever-changing conditions that are characteristic of the modern workplace.

Upon investigating RQ3, concerning the aims and benefits of evaluating the operator’s state, its implications can be used for ergonomic analyses, identifying unsafe postures and movements that could harm an employee’s physical health, quality of life, safety, and productivity. Maintaining correct body posture, especially during physically demanding tasks, can reduce the risk of WMSD, decrease absenteeism, and improve work productivity and safety. Risk assessment tools for industrial production lines provide quantitative information on workstation exposure. It is not negligible that the safety, well-being and performance of the worker are also affected by their mental state. Therefore, the efficient monitoring of physiological parameters is crucial for developing solutions to enhance working activities and environments in industrial production, minimizing work-related stress and discomfort. In this light, the review’s findings offer a starting point for future research aimed at developing integrated systems that not only monitor but also actively enhance both physical and mental well-being in the workplace.

The answers to the three RQs proposed in this survey aim to provide fundamental information to consciously address the integration of ergonomics aspects related to the worker as an individual immersed in the intelligent, connected and collaborative factory. Indeed, such integration, to be effective, cannot be separated from an exploration of the strengths and limitations of current approaches. The results of this study demonstrate the need for an integration of physical and cognitive ergonomics to develop more comprehensive systems that can better support worker well-being in increasingly complex industrial environments. From the results of this survey, it is possible to formulate suggestions for future developments; in this sense, this work provides a series of considerations and information in a synthetic form (tables and charts) to guide researchers in developing integrated systems based on informed choices on instrumentation and methods of analysis. In fact, from all the considerations made, there emerges the need to integrate the two strands, the physical and the cognitive, of the study of worker well-being in the workplace into a single solution capable of uniting and harmonizing the best of the two. These insights provide a foundation for future research aimed at optimizing these integrated approaches and highlight the practical applications of these findings in designing safer and more efficient workplaces. Nevertheless, it is necessary to state that this survey has limitations; in fact, suggestions on the technology to adopt for well-being assessment of the worker are based mainly on the consideration of the most adopted technologies in the proposed studies. The lack of analysis on the actual suitability of these solutions (from several points of view, as highlighted in the Discussion), in terms of devices and methodologies, in the continuous monitoring of worker well-being in industrial workplaces leaves open questions on the best integration strategies of the two branches of ergonomics considered here. In this sense, further studies are needed in which the solutions found to be most widespread and applied in the two contexts are combined and (1) the accuracy of the response, (2) the integrability of the acquisition systems, and (3) the tolerability by the worker in a real industrial scenario are thoroughly assessed. To mitigate this limitation, the authors of this work shared and discussed their direct experience, particularly concerning the acquisition of physiological parameters.

Indeed, even if critical, the human-centric concept of Industry 5.0 cannot disregard the implementation of integrated solutions for physical and cognitive evaluation of the workers’ well-being in the workplace. An example of a fitting application to the context of Industry 5.0 is the integration of the two analyses in the field of HRC, where a system of physical monitoring of the operator via RGB or RGB-D cameras could integrate well with the monitoring of cardiac activity via wearable sensors; the analysis of the data and the extrapolation of indices and parameters via artificial intelligence algorithms, ML and DL, would bring the invaluable advantage of real-time, or at least near real-time, processing of the acquired data, which would guarantee prompt feedback to harmonize the human–robot interaction.

Consequently, the integration of physical and cognitive monitoring appears to be a valid way of taking into account and overcoming the limitations introduced by the two, paving the way for a multi-modal monitoring approach that is truly capable of putting humans at the center of factory design. By doing so, industries can create a future where technology complements human abilities, resulting in a harmonious and productive coexistence between humans and machines in the industrial landscape.

## Figures and Tables

**Table 1:** Keywords adopted for physical perspective.

AI Keywords	Application Keywords	Context Keywords
Artificial Intelligence	Body Tracking	Manufacturing
Machine Learning	Body Recognition	Assembly Line
Deep Learning	Motion Capture	Industrial
	Gesture Recognition	
	Postural Monitoring	
	Ergonomics	

**Table 2:** Keywords adopted for cognitive perspective.

Application Keywords	Context Applications
Mental Ergonomics	Assembly Line
Mental Workload	Manufacturing
Work-Related Stress	Workplace Design
Cognitive Ergonomics	

**Table 3:** Summary of the selected articles. Authors, year of publication, context of application, monitored activity, data collection technology, type of ergonomic parameter (when applicable), and data processing/analysis approach (when applicable) are reported. List of abbreviations: ML/DL Alg. = machine learning/deep learning algorithm, Ergo. = physical or mental ergonomics, Std. ergo. index/Phys. measure = standard ergonomic index/physiological measure.

Authors (Year)	Application Context	Monitored Activity	Acquisition Device	Device Model	ML/DL	Alg.	Ergo.	Std. Ergo. Index/ Phys. Measure
Pławiak et al. (2016) [21]	Hand gestures analysis (body language)	Gesture recognition	Wearable IMUs	DG5 VHand glove (DGTech Engineering Solutions, Bazzano, BO, Italy)	ML	SVM (nu-SVC)	N/A	-
Grzeszick et al. (2017) [22]	Process order picking (warehouse settings)	Pose estimation	Wearable IMUs	Not specified	DL	CNN	N/A	-
Luo et al. (2018) [23]	Collaborative human–robot work in a shared workspace	Human motion prediction	Optical IR marker-based motion capture system	Vicon system (Oxford Metrics, UK)—not specified	ML	GMM	N/A	-
Moya Rueda et al. (2018) [24]	Locomotion gestures and settings in a warehouse (order picking in a logistics scenario)	Human activity recognition	Wearable IMUs	Not specified	DL	CNN	N/A	-
Urgo et al. (2019) [25]	Correctness of movement and safe behaviour in manufacturing mechanical components	Pose estimation and hand/tool tracking	RGB camera	Not specified	Both	HMM CNN (OpenPose)	N/A	-
Chen et al. (2020) [26]	Upper limb assembly action in an industrial setting	Pose estimation	RGB camera	Not specified	DL	YOLO CNN (OpenPose)	N/A	-
Jiao et al. (2020) [27]	Wind turbine blade manufacturing process	Pose estimation and action recognition	RGB camera	Not specified	DL	YOLO SHM STN GCN CNN	N/A	-
Manitsaris et al. (2020) [28]	TV assembly lines, the glassblowing industry, automated guided vehicles, and human–robot collaboration in the automotive assembly lines	Action recognition	(1) RGB camera (2) RGB-D camera	(1) Not specified (2) Not specified	ML	HMM	N/A	-
Phan et al. (2020) [29]	Stiffness along the wrist radial–ulnar deviation during a polishing task	estimation of Human Operators’ Joint Stiffness	(1) Instrumented tool with force/torque sensors; wearable sEMG sensors	(1) ATI mini 40 loadcell and Motion capture VZ4000 (Phoenix Technologies Inc., Vancouver, BC, Canada; (2) BIOPAC MP150 Data Acquisition and Analysis System (BIOPAC Systems Inc., Goleta, CA, USA)	-	-	N/A	-
Xiong et al. (2020) [30]	Cleaning with lying and standing engine block	Action recognition	-	-	DL	CNN	N/A	-
Luipers et al. (2021) [31]	Collaborative work human–robot on the assembly of sensor cases	Human motion prediction	RGB-D camera—Structured light	Kinect (Microsoft Corporation, Redmond, WA, USA)	ML	GP MetaL ANN	N/A	-
Manns et al. (2021) [32]	Manual assembly of extruded aluminium profile in shop floor environments	Action recognition and human motion prediction	Wearable IMUs	(1) XSENS MVN Awinda (Movella Inc., Henderson, NV, USA); (2) Manus Prime II (Movella Inc., Henderson, NV, USA)	DL	LSTM	N/A	-
Niemann et al. (2021) [33]	Order picking and packaging activities	Pose estimation and action recognition	Optical IR marker-based motion capture system	Not specified	DL	tCNN	N/A	-
Papanagiotou et al. (2021) [34]	Collaborative work human–robot on the assembly of LCD TV assembly	Pose estimation and gesture recognition	(1) RGB camera (2) RGB-D camera	(1) GoPro hero9 (San Mateo, CA, USA); (2) Intel-RealSense RGB-D (Santa Clara, CA, USA)—not specified	DL	CNN (OpenPose)		
3DCNN	N/A	-						
Al-Amin et al. (2022) [35]	Assembly of a three-dimensional printer	Action recognition	Wearable IMUs	Myo armbands (Thalmic Labs, Waterloo, ON, Canada)	DL	CNN	N/A	-
Choi et al. (2022) [36]	Human–robot interaction in manufacturing and industrial fields	Pose estimation and Instance Segmentation	RGB-D camera—Time-of-Flight	Azure Kinect (Microsoft Corporation, Redmond, WA, USA)	DL	Mask-RCNN	N/A	-
De Feudis et al. (2022) [37]	Manual industrial assembly/disassembly procedure (power drill)	Pose estimation and hand/tool tracking	RGB-D camera—Time-of-Flight	Azure Kinect	DL	CNN (OpenPose) ArUco YOLO AKBT	N/A	-
Lima et al. (2022) [38]	Developing natural user interfaces to control a robotic arm	Hand state classification	RGB-D camera—Time-of-Flight	Kinect v2	DL	LRCN	N/A	-
Mendes (2022) [39]	Assembly of electric motor in a human–robot collaboration environment	Gesture recognition	Wearable sEMG sensors	(1) Myo armbands; (2) sEMG prototype (Technaid S.L., Alcorcon, Spain)	Both	kNN CNN	N/A	-
Orsag et al. (2023) [40]	Collaborative work human–robot in an industrial environment	Action recognition and human motion prediction	Wearable IMUs	Combination Perception Neuron 32 Edition v2 (Miami, FL, USA)	DL	LSTM	N/A	-
Álvarez et al. (2016) [41]	Instruments for evaluation of the workers’ movements for employee injury and illness reduction (occupational health)	Pose estimation	Wearable IMUs	Model PTUD46 (Directed Perception) (Artisan TG, Champaign, IL, USA)	-	-	Physical	-
Fletcher et al. (2018) [42]	Aircraft wing system installations	Integration of human activity data and ergonomic analysis for digital design modelling and system monitoring	Wearable IMUs	IGS-Bio v1.8 (Animazoo Ltd., Hove, UK)	-	-	Physical	REBA
Golabchi et al. (2018) [43]	Construction job site	Pose estimation and action recognition	RGB camera	Not specified	DL	kNN	Physical	-
Nath et al. (2018) [44]	Warehouse operation (transport and loading an item)	Human activity recognition	Smartphone IMUs	Google Nexus 5X, Google Nexus 6 (Mountain View, CA, USA)	ML	SVM	Physical	-
Grandi et al. (2019) [45]	Workstation layout and the working cycle using digital manufacturing tools (manual assembly of cabin supports on the chassis of a tractor)	Pose estimation	-	-	-	-	Physical	EAWS
Maurice et al. (2019) [46]	Industry-oriented activities (car manufacturing)	Pose estimation	(1) Wearable IMUs; (2) Optical IR marker-based motion capture system; (3) Flexion and force sensor; (4) RGB camera	(1) Xsens MVN Link system (Xsens MVN whole-body Lycra suits; (2) Qualisys motion capture system (Qualisys, Goteborg, Sweden); (3) E-glove (Emphasis Telematics, Athens, Greece); (4) Not specified	Both	HMM CNN (OpenPose)	Physical	EAWS
Conforti et al. (2020) [47]	Manual material handling tasks	Pose estimation	Wearable IMUs	MIMUs MTw (Xsens Technologies)	ML	SVM	Physical	-
Massiris Fernández et al. (2020) [48]	Outdoor working scenarios (performing Marshall signs to an airplane, wall plastering and hammering work activities, tree cutting and drilling job)	Pose estimation	RGB camera	Not specified	DL	CNN (OpenPose)	Physical	RULA
Peruzzini et al. (2020) [49]	Assembly of the air cabin filters	Pose estimation	(1) Multi-parametric wearable sensor for real-time vital parameters monitoring; (2) RGB camera; (3) Optical IR marker-based motion capture system	(1) Zephyr BioHarness 3.0 (Medtronic, Minneapolis, MN, USA); (2) GoPro Hero3; (3) VICON Bonita cameras	-	-	Physical	OWAS, REBA, RULA
Dimitropoulos et al. (2021) [50]	Collaborative work in the elevator production sector	Pose estimation and action recognition	RGB-D camera—Time-of-Flight	Azure Kinect	DL	CNN	Physical	RULA
Mazhar et al. (2021) [51]	Human–robot interaction in social or industrial settings	Pose estimation and gesture recognition	RGB-D camera—Time-of-Flight	Kinect v2	DL	CNN (OpenPose)	Physical	-
Mudiyanselage et al. (2021) [52]	Measurement of muscle activity while performing manual material handling	Pose estimation	Wearable sEMG sensors	Noraxon Mini DTS (Scottsdale, AZ, USA)	ML	Decision Tree SVM KNN Random forest	Physical	-
Ciccarelli et al. (2022) [53]	Posture classification in manufacturing settings (kitchen manufacturing)	Pose estimation	Wearable IMUs	Xsens MTw (Wireless Motion Tracker)	DL	CNN	Physical	RULA
Generosi et al. (2022) [54]	Manufacturing work operations (postures, hand grip types, and body segments)	Pose estimation	RGB camera	iPhone XS (Apple, Cupertino, CA, USA)	DL	CMU CNN (Mediapipe)	Physical	REBA, RULA, OCRA, OWAS
Guo et al. (2022) [55]	Building a virtual scenario for industrial maintenance and assembly process (satellite manufacturing)	Pose estimation	Optical IR marker-based motion capture system	Not specified	-	-	Physical	RULA
Kačerová et al. (2022) [56]	Implementation of ergonomic changes in working position (upper limb loading in the assembly workplace)	Pose estimation	Wearable IMUs	MoCap suit Perception Neuron Studio (Noitom Inc., Miami, FL, USA)	-	-	Physical	-
Lin et al. (2022) [57]	Video-based motion capture and force estimation frameworks for comprehensive ergonomic risk assessment	Pose estimation	(1) RGB camera; (2) Optical IR marker-based motion capture system	(1) GoPro HERO 6; (2) Vicon system—not specified	DL	CNN (OpenPose)	Physical	OWAS, REBA, RULA
Lorenzini et al. (2022) [58]	Kinematic/dynamic monitoring of physical load	Pose estimation	(1) Wearable IMUs; (2) Integrated piezoelectric force platforms; (3) Wearable sEMG sensors	(1) Xsens MVN suit; (2) Kistler force plate (Kistler Holding AG, Winterthur, Switzerland); (3) Delsys Trigno Wireless platform (Delsys Inc., Natick, MA, USA)	-	-	Physical	EAWS
Nunes et al. (2022) [59]	Posture evaluation in industrial settings (automotive assembly line)	Pose estimation	Wearable IMUs	Kallisto IMUs (Sensry Gmbh, Dresden, Germany); MVN Awinda (Xsens, Enschede, The Netherlands)	-	-	Physical	EAWS
Panariello et al. (2022) [60]	Execution of overhead industrial task (an overhead drilling task)	Pose estimation	(1) Optical IR marker-based motion capture system; (2) Integrated Strain Gauge force platforms; (3) Wearable sEMG sensors	(1) SMART DX 6000 (BTS Bioengineering, Garbagnate Milanese, Milano, Italy; (2) P600, BTS Bioengineering; (3) FREEEMG 1000 and 300, BTS Bioengineering	-	-	Physical	RULA
Paudel et al. (2022) [61]	Human body joints estimation for ergonomics (manufacturing settings)	Pose estimation	RGB camera	Not specified	DL	3DMPPE Yolo	Physical	OWAS, REBA, RULA
Vianello et al. (2022) [62]	Online ergonomic feedback to industrial operators with/without interaction with a robot	Pose estimation and action recognition	Wearable IMUs	Xsens MVN suit	DL	VAE	Physical	RULA
Mattsson et al. (2017) [63]	Worker well-being evaluation in manufacturing setting	Mental load measurement	Wearable sensors	Qsensor, Breathing Activity Device, Smart Band 2 (Xiaomi, Beijing, China)	-	-	Mental	HRV, BVP, EDA, skin temperature, respiration
Nardolillo et al. (2017) [64]	Fatigue pattern evaluation in different aged workers (construction, manufacturing)	Mental/physical fatigue	Wearable sensors	Polar heart rate monitor (Polar Electro Oy, Kempele, Finland)	-	-	Mental/physical	HRV
Chen et al. (2017) [65]	Mental workload assessment in contruction workers	Mental load assessment	Wearable sensors	Prototype wearable EEG helmet	-	-	Mental	EEG
Cheema et al. (2018) [66]	Mental load assessment in multitasking activities (HRC)	Mental load assessment	Wearable sensors	Emotiv Epoc+ (Emotiv, San Francisco, CA, USA)	ML	RF	Mental	EEG
Hwang et al. (2018) [67]	Emotional assessment in construction workers	Mental load assessment	Wearable sensors	Not specified	-	-	Mental	EEG
Bommer et al. (2018) [68]	Mental workload assessment in manufacturing settings	Mental load assessment	Near infrared eye tracker	Tobii e T 120 (Tobii, Danderyd, Sweden)	-	-	Mental	eye tracking
D’Addona et al. (2018) [69]	Mental load assessment in manufacturing	Mental load assessment	Passive brain–computer interface (pBCI)	-	-	-	Mental	EEG
Landi et al. (2018) [70]	Mental workload assessment in industrial settings	Mental load assessment for affective robotics	Wearable sensors	Samsung Gear S (Samsung, Suwon, Republic of Korea)	-	-	Mental	HRV
Kosch et al. (2019) [71]	Workload evaluation in manual assembly	Mental load assessment	Wearable sensors	Empatica E4 (Empatica, Boston, MA, USA)	-	-	Mental	EDA
Arpaia et al. (2020) [72]	Mental workload in working environments	Mental load assessment	Wearable sensors	EEG-SMT Olimex (Olimex, Plovdiv, Bulgaria)	Both	SVM, kNN, RF, ANN	Mental	EEG
Bettoni et al. (2020) [73]	Mental workload assessment in manufacturing settings for adaptive HRC	Mental load assessment	Wearable sensors	Polar heart rate monitor, Empatica E4	ML	RF	Mental	HRV, EDA, skin temperature
Lee et al. (2020) [74]	Evaluation of burnout influence on performance in unskilled construction workers	Burnout assessment	(1) Wearable sensors, (2) Wearable IMUs	(1) Zephyr BioHarness3, (2) ActiGraph GT9X (ActiGraph LLC, Pensacola, FL, USA)	-	-	Mental/physical	HR
Papetti et al. (2020) [75]	Integration of physical and mental well-being in industrial settings	Improvement of workers’ well-being	Wearable sensors, eye-tracking glasses	Not specified	-	-	Mental/ physical	HR, HRV, respiration, pupil diameter, eye blink
Van Acker et al. (2020) [76]	Mental workload measurement in manual assembly	Mental load assessment	Eye-tracking glasses	SMI ETG 2w (SensoMotoric Instruments, Teltow, Germany)	-	-	Mental	Pupillometry
Chen et al. (2020) [26]	Mental workload measurement in HRC	Mental load assessment	Eye tracking glasses	Tobii pro glasses2	-	-	Mental	Pupillometry
Digiesi et al. (2020) [77]	Mental load assessment in smart operators (manufacturing activities)	Mental load assessment	ECG monitor	BITalino plugged kit (PLUX wireless biosignals, Lisboa, Portugal)	-	-	Mental	HRV
Mahmad Khairai et al. (2021) [78]	Work stress assessment in assembly line workers	Mental load assessment	Wearable sensors	EmWavePro (HeartMath, Boulder Creek, CA, USA)	-	-	Mental	HRV
Hopko et al. (2021) [79]	Evaluation of mental workload and cognitive fatigue in HRC	Menta load assessment	Wearable sensors	Actiheart 5 (CamNTech, Fenstanton, Cambridgeshire, UK)	-	-	Mental	HRV
Argyle et al. (2021) [80]	Analysis of fatigue and cognitive state in digital manufacturing	Mental load assessment	(1) Wearable sensors, (2) Thermal camera, (3) RGB camera	(1) Artinis Octamon+ (Artinis Medical Systems, Elst, The Netherlands), Zephyr BioHarness3, (2) FLIR A65sc (Teledyne FLIR, Wilsonville, Oregon, USA), (3) Not specified	-	-	Mental	fNIRS, HR, respiration, skin temperature
Brunzini et al. (2021) [81]	Analysis of ergonomics of operators in manual assembly	Workload assessment for HCD of industrial processes	(1) Eye-tracking glasses, (2) Wearable sensors, (3) Wearable IMUs	(1) Tobii glasses2, (2) Empatica E4, (3) Vive trackers 3.0 (HTC, Taoyuan, Taiwan)	-	-	Mental/ physical	RULA, HR, EDA, pupillometry
Bläsing et al. (2021) [82]	Mental workload assessment in manual assembly with assistance system	Mental load assessment	(1) Portable ECG monitor, (2) Eye-tracking glasses	(1) Faros eMotion 180 (BlindSight Gmbh, Fröndenberg, Germany), (2) SMI ETG 2w	-	-	Mental	ECG, eye tracking
Lin et al. (2022) [83]	Mental workload prediction in HRC	Mental workload assessment	Wearable sensors	Smartwatch DTA-S50 (DTAudio, Taiwan)	ML	RF	Mental	GSR, skin temperature, HR, BVP
Mach et al. (2022) [84]	Mental workload measurement for application in industry	Mental load assessment	(1) Wearable sensors, (2) Chest strap	(1) Samsung Gear S3, (2) Garmin premium heart rate monitor (Garmin Ltd., Olathe, KS, USA)	-	-	Mental	HR

**Table 4:** Summary of the parameters adopted for ergonomic assessments. The last column refers to non-standard parameters.

Author	Reference	Year	RULA	REBA	EAWS	OWAS	OCRA	Other
Álvarez et al.	[41]	2016						X
Fletcher et al.	[42]	2018		X				
Golabchi et al.	[43]	2018						X
Nath et al.	[44]	2018						X
Grandi et al.	[45]	2019			X			
Maurice et al.	[46]	2019			X			
Conforti et al.	[47]	2020						X
Massiris Fernández et al.	[48]	2020	X					
Peruzzini et al.	[49]	2020	X	X		X		
Dimitropoulos et al.	[50]	2021	X					
Mazhar et al.	[51]	2021						X
Mudiyanselage et al.	[52]	2021						X
Ciccarelli et al.	[53]	2022	X					
Generosi et al.	[54]	2022	X	X		X	X	
Guo et al.	[55]	2022	X					
Kačerová et al.	[56]	2022						X
Lin et al.	[57]	2022	X	X		X		
Lorenzini et al.	[58]	2022			X			
Nunes et al.	[59]	2022			X			
Panariello et al.	[60]	2022	X					
Paudel et al.	[61]	2022	X	X		X		
Vianello et al.	[62]	2022	X					X

## Data Availability

Data sharing is not applicable.

## Abbreviations

The following abbreviations are used in this manuscript.

**Abbreviations**	**Description**
3DMPPE	3D multiperson pose estimation
ANN	Artificial Neural Network
APM	Action Perception Module
BVP	Blood Volume Pulse
CMU	Carnegie Mellon University
CNN	Convolutional Neural Network
CPM	convolutional pose machine
DHM	Digital Human Model
DL	Deep Learning
DT	Decision Tree
EAWS	Ergonomic Assessment Worksheet
ECG	electrocardiography
EDA	Electrodermal Activity
EEG	Electroencephalography
EIM	Ergonomics Improvement Module
EOG	Electro-oculography
fNIRS	Functional Near-Infrared Spectroscopy
GCN	Graph Convolutional Networks
GMM	Gaussian Mixture Models
GMR	Gaussian Mixture Regression
GP	Gaussian Process
GSR	Galvanic Skin Response
HAR	Human Activity Recognition
HMC	Human Machine Collaboration
HMM	Hidden Markov Model
HRV	Heart Rate Variability
IMA	Industrial Maintenance and Assembly
IMU	Inertial Measurement Units
kNN	K-Nearest Neighbors
KPI	Key Performance Indicator
LPM	Learning and Programming Module
LRCN	Long-term Recurrent Convolutional Network
LSTM	Long Short-Term Memory
MoCap	Motion Capture
OCRA	Occupational Repetitive Actions
oMoCap	Optical Motion Capture
OWAS	Ovako Working Posture Analysis System
PNN	Probabilistic Neural Network
QDA	Quadratic Discriminant Analysis
REBA	Rapid Entire Body Assessment
RF	Random Forest
RMS	Root Mean Square
RULA	Rapid Upper Limb Assessment
sEMG	Surface Electromyography
SHM	Stacked Hourglass Model
SS	State-Space
SSD	Single Shot Detector
StaDNet	Static and Dynamic Gestures Network
STN	Spatial Transformer Networks
SVM	Support Vector Machine
tCNN	Temporal Convolutional Neural Network
TPS	Thin-Plate Splines
VAE	Variational Autoencoder
VR	Virtual Reality
WMSD	Work-Related Musculoskeletal Disorders
XGBoost	eXtreme Gradient Boosting
YOLO	You Only Look Once

## Appendix A

**Table A1:** Summary of the different technologies adopted for the motion acquisition.

Authors	Year	Technology
Álvarez et al. [41]	2016	Wearable IMUs
Pławiak et al. [21]	2016	Wearable IMUs
Grzeszick et al. [22]	2017	Wearable IMUs
Fletcher et al. [42]	2018	Wearable IMUs
Golabchi et al. [43]	2018	RGB camera
Luo et al. [23]	2018	Optical IR marker-based motion capture system
Moya Rueda et al. [24]	2018	Wearable IMUs
Nath et al. [44]	2018	Smartphone IMUs
Maurice et al. [46]	2019	Wearable IMUs
Maurice et al. [46]	2019	Optical IR marker-based motion capture system
Maurice et al. [46]	2019	Force sensors
Maurice et al. [46]	2019	RGB camera
Urgo et al. [25]	2019	RGB camera
Chen et al. [26]	2020	RGB camera
Conforti et al. [47]	2020	Wearable IMUs
Jiao et al. [27]	2020	RGB camera
Manitsaris et al. [28]	2020	RGB camera
Manitsaris et al. [28]	2020	RGB-D camera
Massiris Fernández et al. [48]	2020	RGB camera
Peruzzini et al. [49]	2020	Multi-parametric wearable sensor for real-time vital parameters monitoring
Peruzzini et al. [49]	2020	RGB camera
Peruzzini et al. [49]	2020	Optical IR marker-based motion capture system
Phan et al. [29]	2020	Instrumented tool with force/torque sensors
Phan et al. [29]	2020	Wearable sEMG sensors
Dimitropoulos et al. [50]	2021	RGB-D camera
Luipers et al. [31]	2021	RGB-D camera
Manns et al. [32]	2021	Wearable IMUs
Mazhar et al. [51]	2021	RGB-D camera
Mudiyanselage et al. [52]	2021	Wearable sEMG sensors
Niemann et al. [33]	2021	Optical IR marker-based motion capture system
Papanagiotou et al. [34]	2021	RGB camera
Papanagiotou et al. [34]	2021	RGB-D camera
Al-Amin et al. [35]	2022	Wearable IMUs
Choi et al. [36]	2022	RGB-D camera
Ciccarelli et al. [53]	2022	Wearable IMUs
De Feudis et al. [37]	2022	RGB-D camera
Generosi et al. [54]	2022	RGB camera
Guo et al. [55]	2022	Optical IR marker-based motion capture system
Kačerová [56]	2022	Wearable IMUs
Lima et al. [38]	2022	RGB-D camera
Lin et al. [57]	2022	RGB camera
Lin et al. [57]	2022	Optical IR marker-based motion capture system
Lorenzini et al. [58]	2022	Wearable IMUs
Lorenzini et al. [58]	2022	Force sensors
Lorenzini et al. [58]	2022	Wearable sEMG sensors
Mendes [39]	2022	Wearable sEMG sensors
Nunes et al. [59]	2022	Wearable IMUs
Panariello et al. [60]	2022	Optical IR marker-based motion capture system
Panariello et al. [60]	2022	Force sensors
Panariello et al. [60]	2022	Wearable sEMG sensors
Paudel et al. [61]	2022	RGB camera
Vianello et al. [62]	2022	Wearable IMUs
Orsag et al. [40]	2023	Wearable IMUs

**Table A2:** Link between the device and the monitored activity.

Device	Gesture Recognition	Pose Estimation	Action Recognition	Hand/Tool Tracking	Hand State Classification	Human Motion Prediction	Joint Stiffness Estimation	Human Activity Recognition	Digital Design Modelling
w IMUs	X	X	X			X		X	X
RGB-D MDPI:	X	X	X	X	X	X			
oMoCap		X	X			X			
RGB	X	X		X					
w sEMG	X	X							
Smartph. IMUs								X	
w IMUs, oMoCap, Flex./Force sens., RGB	X								
Multi-par. w sensor, RGB, oMoCa p	X								
Force/Torque sens.							X		
w IMUs, Force sens., w sEMG	X								
oMoCap, Force sens. W sEMG	X								

**Table A3:** Link between the device and the monitored activity.

		ML									DL											
**Author**	**Year**	**ANN**	**Decision Tree**	**GMM**	**GP**	**HMM**	**kNN**	**Meta Learning**	**Random Forest**	**SVM**	**3D MPPE**	**ArUco**	**AKBT**	**DL Mod. CMU**	**CNN**	**GCN**	**LRCN**	**LSTM**	**SHM**	**STN**	**VAE**	**YOLO**
Pławiak et al. [21]	2016									X												
Grzeszick et al. [22]	2017														X							
Golabchi et al. [43]	2018						X															
Luo et al. [23]	2018			X																		
Moya Rueda et al. [24]	2018														X							
Nath et al. [44]	2018									X												
Maurice et al. [46]	2019					X									X							
Urgo et al. [25]	2019					X									X							
Chen et al. [26]	2020														X							X
Conforti et al. [47]	2020									X												
Jiao et al. [27]	2020														X	X			X	X		X
Manitsaris et al. [28]	2020					X																
Massiris Fernández et al. [48]	2020														X							
Xiong et al. [30]	2020														X							
Dimitropoulos et al. [50]	2021														X							
Luipers et al. [31]	2021	X			X			X														
Manns et al. [32]	2021																	X				
Mazhar et al. [51]	2021														X							
Mudiyanselage et al. [52]	2021		X				X		X	X												
Niemann et al. [33]	2021														X							
Papanagiotou et al. [34]	2021														X							
Al-Amin et al. [35]	2022														X							
Choi et al. [36]	2022														X							
Ciccarelli et al. [53]	2022														X							
De Feudis et al. [37]	2022											X	X		X							X
Generosi et al. [54]	2022													X	X							
Lima et al. [54]	2022																X					
Lin et al. [57]	2022														X							
Mendes [39]	2022						X								X							
Paudel et al. [39]	2022										X											X
Vianello et al. [62]	2022																				X	
Orsag et al. [40]	2023																	X				
